# The invariant ion-acoustic waves in the plasma

**DOI:** 10.1038/s41598-022-25233-y

**Published:** 2022-12-16

**Authors:** E. Saberian

**Affiliations:** Department of Physics, Faculty of Basic Sciences, University of Neyshabur, Neyshabur, 9319774446 Iran

**Keywords:** Astrophysical plasmas, Nonlinear phenomena

## Abstract

The space plasmas have been found empirically to be separated into those residing far from the classical thermal equilibrium and those residing near equilibrium. The modern formalism of the kappa distributions explains this distinction under the value of the kappa index, the intensive parameter that characterizes thermodynamics together with temperature. Recent studies have suggested that by defining an invariant kappa index as the zero dimensionality spectral index, $\kappa _{0}$, which is independent of the dimensionality, the degrees of freedom, or the numbers of particles, one may separately consider the physical and thermodynamic feature of the kappa index in space plasmas by utilizing $\kappa _{0}$. This study extends the mentioned idea to the ion-acoustic waves (IAWs) in the astrophysical plasmas in order to deriving an invariant formalism for the IAWs including the pure thermodynamic features of the background particles. This paper is based on the kinetic theory formalism and the hydrodynamic fluid description for extracting the characteristics of the invariant IAWs. Relying on the Vlasov–Poisson equations, considering a low-frequency band for the weakly damped ion oscillations, we have derived the most generalized formalism of the ion-sound speed in space plasmas in terms of the extended polytropic indices of the plasma species, $\gamma _{j}$, and also the generalized formalism of Landau damping for the invariant IAWs in terms of $\kappa _{0}$, wavelength, and temperatures of the plasma species. In the hydrodynamic description, we have normalized the fluid parameters in terms of the generalized quantities, including the extended formulations of the ion-sound speed and Debye length. Then, by using the perturbation expansion in linear and nonlinear regimes, we may find some other issues in the formalism of the invariant IAWs, such as the effect of the perturbed potential degrees of freedom, $d_{\Phi }$, the isothermal/extended phase speed of the IAWs, and the combined effects of the wave steepening and dispersion of ion waves. We have also derived a generalized KdV equation and its solitary wave solutions in an invariant formalism. Based on the empirical evidences in space plasmas, the far-equilibrium plasmas are characterized by $0<\kappa _{0}<1$ ($0<\gamma _{j}<0.5$), while the near-equilibrium plasmas are labeled with $\kappa _{0}>1$ ($0.5<\gamma _{j}<1$). We have numerically analyzed our solutions from the anti-equilibrium states at $\kappa _{0}\rightarrow 0$ ($\gamma _{j}\rightarrow 0$) towards the equilibrium states at $\kappa _{0}\gg 1$ ($\gamma _{j}\rightarrow 1$). Our theoretical study provides strong evidence, for the first time, about the distinction of plasmas under the value of the kappa index. Our analysis confirms the distinction of the involved IAWs diagrams in the two mentioned regions, where the transition from far-equilibrium states to the near-equilibrium states may occur in the vicinity $\kappa _{0}\sim 1$ ($\gamma _{j}\sim 0.5$), denoting the escape state of the evolution.

## Introduction

Understanding the features and properties of the ion waves in space plasmas and laboratory-produced plasmas is one of the oldest and yet fundamental problems in plasma physics. The ion waves are low-frequency oscillations around a few kHz, which may propagate and transfer the energy in space if they have been excited by a source of energy. These modes have similarities with the sound waves in ordinary gases (e.g., the earth’s atmosphere at lower amplitudes) as they are the longitudinal oscillations and produce the rarefaction/compression in the plasma environment, but they have some differences from the ordinary sound waves: (a) the ions oscillate in phase in the wavefronts instead of the neutral molecules; (b) the electromagnetic forces are dominant instead of the head-on collision forces by the neutral atoms, so they are known as the ion-acoustic waves (IAWs). The essential features of the IAWs such as the propagation, the dispersion relation, Landau damping, etc., have been widely discussed in many textbooks and literature (see e.g.,^1–7^). However, there are yet some new and interesting aspects of the IAWs that have motivated us re-studying this problem by using more advanced formalisms that cover some subtle features of the IAWs. It may provide a deeper insight into the formalism of the IAWs and their features in the astrophysical plasmas.

In recent years, applying the *q*-deformed stationary state in the Tsallis non-extensive statistics^8,9^ or the $\kappa$ distribution formalism^10^ have been becoming very common for studying the plasma waves and the other aspects of the plasmas (see e.g.,^11–14^). The main idea behind applying these formalisms is providing the extended solutions, where we usually examine the deviation of our solutions from thermal equilibrium state by using the role of the spectral indices *q* or $\kappa$.

Here, we mention some studies on the electrostatic waves and oscillations using the Tsallis non-extensive statistics. The plasma oscillations in a collision-less electron-ion plasma have been studied by Lima et al.^15^ and also by Chen and Li^16^ in the context of the non-extensive statistics, where the analytical formulas for the Bohm-Gross waves and their Landau damping derived and discussed. Furthermore, Liyan and Jiulin^17^ have investigated the dispersion relation and Landau damping of IAWs in a collision-less magnetic-field-free plasma, where a *q*-exponential distribution used in one dimension. There, it proved that the non-extensive parameter *q* is related to the temperature gradient and the potential energy. In the similar studies, the ion plasma waves in a pure pair-ions plasma consisting of fullerenes $C_{60}^{\pm }$^18,19^ or correspondingly plasma oscillations in a collisionless electron-positron plasma have studied by applying a 1-dimensional *q*-exponential distribution function^20–22^. The nonlinear features of the IAWs had also discussed by some authors by using the canonical *q*-exponential distribution. For example, the arbitrary amplitude ion-acoustic solitary waves (IASWs) have addressed in a two-component plasma by considering a *q*-exponential distribution for the electrons^23^; or the ion-acoustic double layers in a two-component plasma have studied in the context of the *q*-nonextensive electron distribution^24^, and many others.

In the mentioned studies (and many similar papers), the primary version of *q*-exponential probability distribution (the ordinary or old formalism) have used, where it may be constructed by maximizing Tsallis *q*-entropy under some constraints^25,26^. The ordinary version of the canonical probability distribution had some physical inconsistencies that have been covered later by introducing the notion of the escort probability distribution (the modern formalism) and some other constraints^27^.

It has been proved that the spectral indices *q* or $\kappa$ involve an inherent dimensional dependency to the numbers of degrees of freedom^28,29^. For example, the connection of the kappa index with the number of degrees of freedom is so that the difference $\kappa _{d}-\frac{d}{2}$ is constant and independent of *d*, where *d* is the number of degrees of freedom and $\kappa _{d}$ is *d*-dimensional spectral index. However, the fact is that the indices *q* and $\kappa$ are not invariant, but they depend on the dimensionality. In this regard, we have recently discussed the dependency of the plasma oscillations on the numbers of degrees of freedom (dimensionality) involved in the spectral indices (*q* or $\kappa$) of the non-extensive statistical mechanics^30^.

In the supplementary materials of this study, the formalisms of modern canonical probability distributions and their dependency on the *d*-dimensional spectral indices ($q_{d}$ or $\kappa _{d}$) have presented. There, we have also introduced the formalism of the invariant canonical distribution. In this formalism, by defining an invariant kappa index as of zero dimensionality spectral index, $\kappa _{0}$, which is independent of the dimensionality, the degrees of freedom, or the numbers of particles, one may separately consider the physical and thermodynamic features of the kappa index in space plasmas by utilizing $\kappa _{0}$. It’s the main significance of this formalism in space or lab plasma. Note that the *d*-dimensional index $\kappa _{d}$ depends on the invariant index $\kappa _{0}$ by the relation $\kappa _{d}=\kappa _{0}+\frac{d}{2}$. Some evidence shows the success of this formalism in studying the specific phenomena in space plasmas^31–36^. In summary, the modern version of the probability distribution has some advantages, as opposed to the old versions, such as it is independent of an energy level; it provides correct and consistent partition of the system’s internal energy to the subsystem’s partial internal energies, and it consistent with a meaningful temperature^37^. Another feature (advantage) of the modern canonical distribution is that, by this formalism, the equipartition of degrees of freedom holds in the same way as in the classical case, i.e., $\frac{1}{2}m \left< \vec {u}^{2}\right>=\frac{d}{2}k_{B}T$, where *d* is the number of degrees of freedom. While considering the equipartition of the energy using the old versions of the canonical distribution, one may derive some additional coefficients in terms of $\kappa$ or *q* indices.

Fortunately, the kappa distributions are connected to the zeroth law of thermodynamic and the thermal equilibrium, the concept that reveals the thermodynamic definition of temperature. It has revealed that not only the kappa distributions are consistent with the concept of thermal equilibrium—so they are allowed to be parameterized by temperature—but also the most generalized formalism consistent with the thermal equilibrium is that of kappa distributions^38^. It has proved that when particle systems reach the thermal equilibrium, we have two thermodynamic integrals corresponding to the temperature and the kappa index, as two independent intensive thermodynamic quantities. Note that no correlations exist among the particles in thermal equilibrium via the Maxwell-Boltzmann distribution (the classical thermal equilibrium) while the kappa distributions correspond to the generalized thermal equilibrium, where correlations may exist^38^.

In this paper, we want to study the celebrated problem of the IAWs by using the invariant kappa distribution formalism labeled with an invariant kappa index as of the zero dimensionality spectral index, $\kappa _{0}$. Our strategy in this paper is as follows: First, we will apply a kinetic theory formalism based on the Vlasov-Poisson equations at the low-frequency band of the ion waves, where we will derive the generalized formulations of the dispersion relation and Landau damping of IAWs in terms $\kappa _{0}$; Then, the physical and thermodynamic features of the IAWs will be studied in terms of the extended polytropic indices of thermodynamic evolutions; In the next approach, we will use a hydrodynamic formalism for studying the linear/nonlinear characteristics of the invariant IAWs and the other missing issues, where we will derive a generalized KdV equation and its solitary wave solutions of the invariant IAWs; Finally, we will summarize the concluding remarks.

## The model equations

- *The Vlasov-Poisson equations*
  We assume that at the time $t=0$, a perturbation occurs in a field-free plasma. Then the initial distribution for the species $\alpha$ with mass $m_{\alpha }$, temperature $T_{\alpha }$ and thermal speed $\theta _{\alpha }$ may be described as $P_{\alpha }(\vec {r},\vec {u};\theta _{\alpha };t=0)=P_{\alpha ,0}(\vec {u};\theta _{\alpha })+P_{\alpha ,1}(r\vec {},\vec {u};\theta _{\alpha },t=0)$, where $P_{\alpha ,0}(\vec {u};\theta _{\alpha })$ is the unperturbed time-independent stationary state of the plasma and $P_{\alpha ,1}(\vec {r},\vec {u};\theta _{\alpha },t=0)$ is the corresponding perturbation about the initial state, where $P_{\alpha 0}\ll P_{\alpha 1}$. The time evolution of $P_{\alpha ,1}(\vec {r},\vec {u};\theta _{\alpha },t=0)$ for the small amplitude perturbations may be described by the linearized Vlasov and Poisson equations as follows
  1a $$\begin{aligned}{} & {} \frac{\partial P_{\alpha 1}}{\partial t}+\vec {u} \cdot \frac{\partial P_{\alpha 1}}{\partial \vec {r}} +\frac{q_{\alpha }}{m_{\alpha }} \frac{\partial \phi _{1}}{\partial \vec {r}} \cdot \frac{\partial P_{\alpha 0}}{\partial \vec {u}}\;=\;0, \end{aligned}$$
  1b $$\begin{aligned}{} & {} \nabla ^{2} \phi _{1} =-4\pi \sum _{\alpha } n_{\alpha }q_{\alpha } \int P_{\alpha 1}\, {\text{d}} {\vec {u}}, \end{aligned}$$
  where $\phi _{1}(\vec {r},t)$ is the electrostatic potential produced by the perturbation, $\alpha$ stands for the plasma species ($\alpha =e,i$), and $q_{\alpha }$ and $n_{\alpha }$ denote the charge and number density of the species $\alpha$. By this model equations, we may extract the response dielectric function $D(\vec {k},\omega )$ of the plasma to a typical perturbation, related to the assumed stationary state of the plasma, where it may give the related dispersion relation and Landau damping rate of plasma normal modes. Here, $\omega$ and $\vec {k}$ are the wave frequency and wave vector of the plasma normal modes, respectively.
- *The hydrodynamic equations*
  For discussing the linear/nonlinear characteristics of the invariant IAWs by using the perturbation technique, we need the set of hydrodynamic equations in a warm plasma as follows
  2a $$\begin{aligned}{} & {} \frac{\partial n_{i}}{\partial t} + \frac{\partial (n_{i}v_{i}) }{\partial x}=0, \end{aligned}$$
  2b $$\begin{aligned}{} & {} \left( \frac{\partial v_{i}}{\partial t} + v_{i} \frac{\partial v_{i}}{\partial x} \right) = -\frac{ Z_{i}e}{ m_{i}} \frac{\partial \phi }{\partial x} - \frac{1}{ m_{i} n_{i}} \frac{\partial p_{i}}{\partial x}, \end{aligned}$$
  2c $$\begin{aligned}{} & {} \frac{\partial p_{i}}{\partial t} + v_{i} \frac{\partial p_{i}}{\partial x} + \gamma _{i} \; p_{i} \frac{\partial v_{i}}{\partial x}=0, \end{aligned}$$
  2d $$\begin{aligned}{} & {} \varepsilon _{0} \frac{\partial ^{2}\phi }{\partial x^{2}}=-e(Z_{i}n_{i}-n_{e}), \end{aligned}$$
  where without loss of the generality, we have considered the wave vector in direction to the *x*-axis. Here, $n_{i}$, $v_{i}$, and $p_{i}$ are the number density, fluid velocity, and the pressure of the ions, respectively, and $n_{e}$ is the number density of electrons in the propagation of the ion weaves. Note that the electronic distribution is a function of the potential as $n_{e}(\phi )$. In our notation, $Z_{i}$ denotes the number of charges of the ions that depends on the atomic number of the ions, e.g., $Z_{i}=1$ denotes a Hydrogen plasma ($H^{+1}$ ions) and $Z_{i}=2$ denotes a Helium plasma ($He^{+2}$ ions). Note that the coefficient $\gamma _{i}$ in the pressure evolution equation is the polytropic index of the ions in compression/rarefaction of the longitudinal ion waves. For example, in the propagation of the ion waves in one dimension ($d_{i}=1$) we have $\gamma _{i}=3$, or in three dimensions ($d_{i}=3$) we have $\gamma _{i}=\frac{5}{3}$.
- *The invariant kappa distribution*
  The best choice for studying the pure characteristics of the IAWs is utilizing the invariant kappa index $\kappa _{0}$, which is independent of the dimensionality, the degrees of freedom, or the numbers of particles, and also it contains the physical and thermodynamic features of the kappa index. The general formalisms of the escort canonical distributions and the features of the invariant spectral indices have been given in the supplementary material. The complete expression of *d*-dimensional canonical probability distribution for the species $\alpha$ in terms of $\kappa _{0}$ is given as follows
  3 $$\begin{aligned} P_{\alpha }(\vec {u};\theta _{\alpha };\kappa _{0},d) = \left( \pi \kappa _{0} \theta _{\alpha }^{2}\right) ^{-\frac{d}{2}} \frac{\Gamma (\kappa _{0}+1+\frac{d}{2})}{\Gamma (\kappa _{0}+1)}\cdot \left[ 1+\frac{1}{\kappa _{0}} \cdot \frac{u^{2}}{\theta _{\alpha }^{2}} \right] ^{-\kappa _{0}-1-\frac{d}{2}}, \end{aligned}$$
  where $\theta _{\alpha }=(\frac{2 k_{B}T_{\alpha }}{m_{\alpha }})^{\frac{d}{2}}$ is the *d*-dimensional classical thermal speed of species $\alpha$ with the mass $m_{\alpha }$ and temperature $T_{\alpha }$. We remind that $P_{\alpha }(\vec {u};\theta _{\alpha };\kappa _{0},d)$ satisfies the equipartition of energy as $\left<\frac{1}{2}m_{\alpha } u^{2}\right>=\frac{d}{2}k_{B}T_{\alpha }$, in the same exact way of the classical distribution. In this formalism, the energetic particles may be distributed in one of two sub-regions, i.e., the particles far from thermal equilibrium states with the spectral indices $\kappa _{0}<1$; and the particles near the equilibrium states with the spectral indices $\kappa _{0}>1$. Here, the stationary state with the spectral index $\kappa _{0}=1$ denotes the escape state of the plasma, where the system can escape from the far-equilibrium toward the near-equilibrium regions^28^. We also remind that two asymptotic limits in this notation are the equilibrium state ($\kappa _{0}\rightarrow \infty$) and the anti-equilibrium state ($\kappa _{0}\rightarrow 0$), where the distribution function collapses. In this formalism, the velocity distribution functions with lower $\kappa _{0}$ indices are related to the distributions with more supra-thermal particles (high energy tails), so they have been distributed in a wider range of velocities.

## Results and discussion

### Dispersion relation: the generalized ion-sound speed

For solving the linearized Vlasov-Poisson equations, with no loss of generality, we may consider the wave vector $\vec {k}$ to be in the direction of the *x*-axis and use the 1-dimensional canonical distribution (by choosing $d=1$), where $\vec {r}\rightarrow x$ and $\vec {u}\rightarrow u_{x}$. Note that in an ordinary electron-ion plasma, both the electrons and ions contribute to the dynamics of the IAWs. So, the real part of the dielectric function may be written as

4 $$\begin{aligned} D_{r}(k,\omega _{r})=1-\sum _{\alpha }\frac{\omega _{pe}^{2}}{k^{2}} \; {\mathcal {P}} \int \frac{\frac{\partial P_{e0}}{\partial u_{x}}}{u_{x}-\frac{\omega _{r}}{|k|}}\,\textrm{d}u_{x} +\sum _{\alpha }\frac{\omega _{pi}^{2}}{k^{2}} \; {\mathcal {P}} \int \frac{\frac{\partial P_{i0}}{\partial u_{x}}}{u_{x}-\frac{\omega _{r}}{|k|}}\,\textrm{d}u_{x}, \end{aligned}$$

where $\omega _{pe(i)}=\left( \frac{4\pi n_{\infty ,e(i)}e^{2}}{m_{e(i)}}\right) ^{\frac{1}{2}}$ is the electronic (ionic) plasma frequency, and $n_{\infty ,e(i)}$ is the number density of the electrons (ions) at the unperturbed state (at the infinity). In our notation, the subscripts *r* and *i* denote the real and imaginary parts of the parameters, respectively, and ${\mathcal {P}}$ denotes the Cauchy principal value. Here, $P_{e(i)0}$ is the 1-dimensional stationary state of the electrons (ions), where we consider the formalism of the invariant kappa distribution for them as given in Eq. (3), where $d=1$.

The phase speed of IAWs with $\frac{T_{i}}{m_{i}}\ll \frac{T_{e}}{m_{e}}$ lies between thermal velocities of the ion and electron as $\theta _{i}<v_{\phi } \ll \theta _{e}$, where $v_{\phi }$ denotes the phase speed and $\theta _{i(e)}=\sqrt{\frac{2 k_{B}T_{i(e)}}{m_{i(e)}}}$ stands for the ion (electron) thermal speed. Then, by appropriate Taylor expanding of the integrands in Eq. (4), and by calculating the Cauchy principal values we may find the real part of the dielectric function as follows

5 $$\begin{aligned} D_{r}(k,\omega _{r})=1+\left( \frac{\kappa _{0}+1}{\kappa _{0}}\right) \cdot \frac{1}{(k\lambda _{De})^{2}} -\frac{\omega _{pi}^{2}}{\omega _{r}^{2}} -\frac{\omega _{pi}^{2}}{\omega _{r}^{4}} \cdot \left( \frac{3k_{B}T_{i}}{m_{i}}\right) k^{2}, \end{aligned}$$

where $\lambda _{De(i)}=\left( \frac{k_{B}T_{e}}{4\pi n_{\infty ,e(i)}e^{2}}\right) ^{\frac{1}{2}}$ is the electronic (ionic) Debye screening length. Here, we have used the parametric relation $\theta _{i(e)}^{2}=2\omega _{pe(i)}^{2} \cdot \lambda _{De(i)}^{2}$ in our simplifications. Note that the last term of Eq. (5) may be more simplified as $3\frac{\omega _{pi}^{4}}{\omega _{r}^{4}} (k\lambda _{Di})^{2}$, but we have retained it in terms of the ion temperature for our next plan. The methods for calculation of the real part of the dielectric function, i.e., Eq. (5), is given in the supplementary material.

The dispersion relation of the invariant IAWs may be derived by solving the relation $D_{r}(k,\omega _{r})=0$. The full solution of $D_{r}(k,\omega _{r})=0$ gives a quartic equation in terms of $\omega _{r}$ as follows

6 $$\begin{aligned} a \; \omega _{r}^{4}+b \; \omega _{r}^{2}+c=0, \end{aligned}$$

where the coefficients *a*, *b* and *c* are given as follows

7 $$\begin{aligned} a=\frac{(k\lambda _{De})^{2}+\frac{\kappa _{0}+1}{\kappa _{0}}}{(k\lambda _{De})^{2}}, \; \; b=-\omega _{pi}^{2}, \; \; c=-\omega _{pi}^{2}\left( \frac{3k_{B}T_{i}}{m_{i}}\right) k^{2}. \end{aligned}$$

The solution of Eq. (6) may be simply derived in terms of $\omega _{r}^{2}$ by using the quadratic formula as $\omega _{r}^{2}=\frac{-b\pm \sqrt{b^{2}-4ac}}{2a}$. Then, by algebraic simplifying and rearranging the solution, we may find the following expression

8 $$\begin{aligned} \frac{\omega _{r}^{2}}{k^{2}}=\frac{k_{B}T_{e}}{m_{i}} \left\{ \frac{1\pm \sqrt{1+12\left[ (k\lambda _{De})^{2}+\frac{\kappa _{0}+1}{\kappa _{0}}\right] \cdot \frac{T_{i}}{T_{e}}}}{2\left[ (k\lambda _{De})^{2}+\frac{\kappa _{0}+1}{\kappa _{0}}\right] } \right\} , \end{aligned}$$

where we have used $\omega _{pi}^{2}\cdot \lambda _{De}^{2} = \frac{k_{B}T_{e}}{m_{i}}$. Note that the minus branch is not accepted because it doesn’t satisfy a real phase speed.

By considering $\frac{T_{i}}{T_{e}}\ll 1$ in an ordinary electron-ion plasma and by using the celebrated binomial approximation as $(1+\varepsilon )^{n}\approx 1+n\varepsilon$, where $\varepsilon \ll 1$, the positive (acceptable) branch of Eq. (8) may be simplified as

9 $$\begin{aligned} \frac{\omega _{r}^{2}}{k^{2}}=\frac{k_{B}T_{e}}{m_{i}} \left\{ \frac{1+1+6\left[ (k\lambda _{De})^{2}+\frac{\kappa _{0}+1}{\kappa _{0}}\right] \cdot \frac{T_{i}}{T_{e}}}{2\left[ (k\lambda _{De})^{2}+\frac{\kappa _{0}+1}{\kappa _{0}}\right] } \right\} , \end{aligned}$$

and then, we may derive the generalized dispersion relation of the invariant IAWs as follows

10 $$\begin{aligned} \frac{\omega _{r}}{k}= \left( \frac{k_{B}T_{e}}{m_{i}} \cdot \frac{1}{(k\lambda _{De})^{2}+\frac{\kappa _{0}+1}{\kappa _{0}}}+\frac{3k_{B}T_{i}}{m_{i}} \right) ^{\frac{1}{2}}. \end{aligned}$$

One of the interesting results of Eq. (10) is providing an alternative context for revisiting the generalized formulation of the ion-sound speed in a kappa distributed plasma, besides the hydrodynamics approach as shown in Ref.^36^. By using the definition of the sound speed as $c_{s}=\lim _{k\rightarrow 0}\frac{\omega _{r}}{k}$, we may find a generalized formula for the ion-sound speed of the space plasmas in terms of the invariant kappa index as follows

11 $$\begin{aligned} c_{s,\kappa _{0}}=\left( \frac{\kappa _{0}}{\kappa _{0}+1} \cdot \frac{k_{B}T_{e}}{m_{i}} +\frac{3k_{B}T_{i}}{m_{i}} \right) ^{\frac{1}{2}}. \end{aligned}$$

We may rewrite this solution in terms of the generalized polytropic index of kappa distributed plasmas^39^, where its formalism is

12 $$\begin{aligned} \gamma =\frac{\kappa _{0}+\frac{1}{2}d_{\Phi }}{\kappa _{0}+\frac{1}{2}d_{\Phi }+1}, \end{aligned}$$

where $d_{\Phi }$ is the potential degrees of freedom in the plasma, with the definition as the ensemble average of the potential energy, $\Phi$, i.e., $d_{\Phi }=\frac{<\Phi >}{k_{B}T}$. It has emphasized that the polytropic index satisfies the thermodynamic evolutionary relationship, where $p({\bar{r}})$ is the thermal pressure and $n(\vec {r})$ denotes the number density of kappa distributed particles^39^.

Here, we have implicitly neglected the potential energy in the canonical probability distribution of Eq. (3). So, the potential degrees of freedom is $d_{\Phi }=0$, when other source of potential energy in the plasma don’t exist, such as the electromagnetic and gravitational fields. Then, the polytropic index of kappa distributed particles is given only in terms of the spectral index $\kappa _{0}$ as $\gamma =\frac{\kappa _{0}}{\kappa _{0}+1}$. In the next section, we will employ a perturbation theory for deriving the nonlinear aspects of the invariant IAWs, where the potential degrees of freedom via the perturbation appear in our formalism.

In the propagation of the IAWs, the inertial ions oscillate in one-dimensional compressions/rarefactions along with the propagation of the wave, as we have considered in our model equations by choosing $d=1$. It implies that the polytropic index of the ions takes the value $\gamma _{i}=\frac{d_{i}+2}{d_{i}}=3$, where $d_{i}=1$ is the number of degrees of freedom for the ions. In our kinetic model, the solution of the Vlasov-Poisson equations for the IAWs implies correctly the adiabatic index $\gamma _{i}=3$ for the ions (see, e.g., Eqs. (10) and (11)). On the other hand, the inertialess electrons are pulled along with the ion waves and they contribute to the screening of the electric fields arising from the bunching of the ions. Here, the thermal distribution of the electrons is the invariant kappa formalism. As we mentioned, the polytropic index of the electrons takes the values $\gamma _{e}=\frac{\kappa _{0}}{\kappa _{0}+1}$ in terms of $\kappa _{0}$.

Then, our generalized formulation for the ion-sound speed may be re-written in terms of the polytropic indices of the electrons and ions as follows

13 $$\begin{aligned} c_{s}(\gamma _{e},\gamma _{i})=\left( \frac{\gamma _{e}k_{B}T_{e}}{m_{i}} +\frac{\gamma _{i}k_{B}T_{i}}{m_{i}} \right) ^{\frac{1}{2}}. \end{aligned}$$

This result confirms the recent finding of the generalized ion-sound speed in space and astrophysical plasmas^36^, where the hydrodynamic equations had employed. The main idea is that the ion-sound speed is a sensitive function of the thermodynamic state of the plasma and it may vary between the near/far-from-equilibrium states of the plasma.

**Figure 1:**
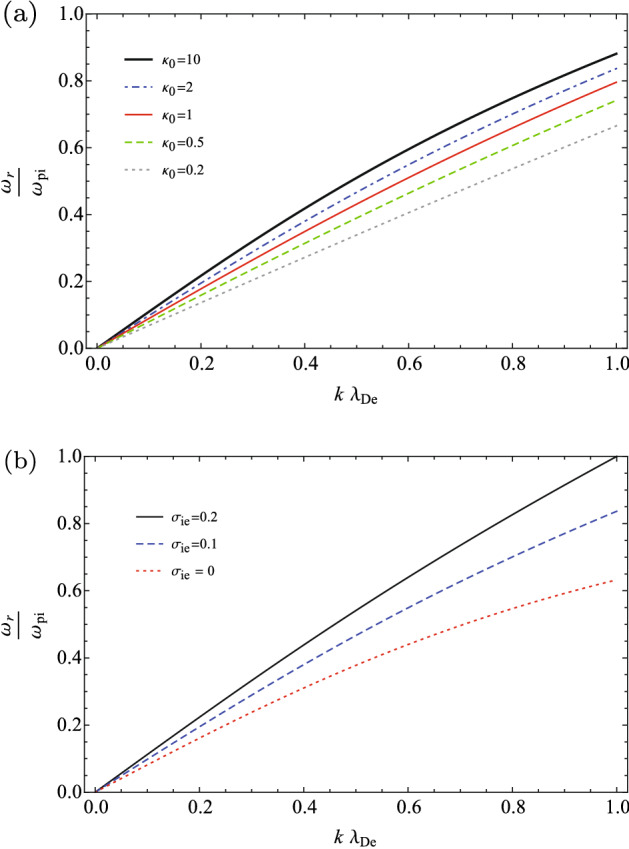
Dispersion relation of IAW: (**a**) for some typical invariant spectral indices when $\sigma _{ie}=0.1$ and $\gamma _{i}=3$; (**b**) for some typical values of the fractional ion to electron temperature when $\kappa _{0}=2$ and $\gamma _{i}=3$.

The dispersion relation of Eq. (10) may be re-written in terms of the normalized parameters as

14 $$\begin{aligned} \omega ^{'}_{r}(k^{'})= k^{'} \left( \frac{1}{{k^{'}}^{2}+\frac{\kappa _{0}+1}{\kappa _{0}}}+\gamma _{i}\sigma _{ie} \right) ^{\frac{1}{2}}, \end{aligned}$$

where $\omega {'}_{r}=\frac{\omega _{r}}{\omega _{pi}}$ is the frequency of IAWs normalized to the ion plasma frequency, $k^{'}=\frac{k}{k_{De}}$ is the normalized wave number, where $k_{De}=\frac{1}{\lambda _{De}}$, and $\sigma _{ie}=\frac{T_{i}}{T_{e}}$ is the fractional ion to electron temperature. Here, we have used $\gamma _{i}$ as the general polytropic index of the ions to improve the generality of our formalism. Then, we may compare the dispersion relation of IAWs in different thermodynamics states of typical space plasmas, where the sub-isothermal processes may occur, or when the thermodynamics processes are very close to the anti-equilibrium state, or when they are very close to the thermal equilibrium, or even when the transitions between the near/far-equilibrium states take place. We have depicted the variation of dispersion relation in terms of the spectral index $\kappa _{0}$ (equivalently in terms of $\gamma _{e}$), and also in terms of $\sigma _{ie}$, as given in two panels of Fig. 1.

In panel (a) in Fig. 1, we have considered a fixed fractional ion to electron temperature as $\sigma _{ie}=0.1$, the polytropic index of the ions as $\gamma _{i}=3$ (corresponding to the one-dimensional compressions/rarefactions of the ion waves), and some invariant spectral indices from the far-equilibrium regions ($0<\kappa _{0}<1$) to the near-equilibrium regions ($\kappa _{0}>1$).

Our analysis shows that the ion-sound speed (the slope of the tangent line to the dispersion relation at the point $k\rightarrow 0$) increases for the higher $\kappa _{0}$ indices, where it tends to the maximum phase speed of IAWs at the Maxwellian limit $\kappa _{0}\rightarrow \infty$, or equivalently at the isothermal limit $\gamma _{e}\rightarrow 1$. This picture confirms the results in Ref.^36^, where the ion-sound speed had analyzed using the hydrodynamics formalism. In terms of the generalized polytropic index ($\gamma _{e}=\frac{\kappa _{0}}{\kappa _{0}+1}$), the chosen polytropic indices in Fig. 1 are respectively $\gamma _{e}=0.17$ when $\kappa _{0}=0.2$, $\gamma _{e}=0.33$ when $\kappa _{0}=0.5$, $\gamma _{e}=0.5$ when $\kappa _{0}=1$ (the escape state), $\gamma _{e}=0.67$ when $\kappa _{0}=2$, and $\gamma _{e}=0.91$ when $\kappa _{0}=10$.

We have to note that the selected kappa and polytropic indices for the electrons/ions have been chosen to be close to the observational data in space physics environments. For example, the spectral index in the ambient solar wind (SW) regions is reported to be on the order $\kappa _{3}\sim 1.5 \pm 0.1$ ($\kappa _{0}\sim 0$)^40^, where the extended polytropic index is very close to the anti-equilibrium state $\gamma _{e}\sim 0$.

On the other hand, the kappa index in the outer heliosphere regions has reported to be on the order $\kappa _{3}\sim 1.63 \pm 0.05$ ($\kappa _{0}\sim 0.13$)^41^, where the polytropic index is on the order $\gamma _{e}\sim 0.11$; or the kappa index in the inner heliosheath (IH) regions has been reported to be on the order $\kappa _{3}\sim 1.75 \pm 0.10$ ($\kappa _{0}\sim 0.25$)^42^, where the extended polytropic index is on the order $\gamma _{e}\sim 0.2$. These are examples of the far-equilibrium regions, where $\gamma _{e}<1$, and the related thermodynamic processes are sub-isothermal.

Moreover, the spectral kappa index in the slow solar wind $\mathrm {e^{-}}$ (Ulysses) plasmas has reported to be on the order $\kappa _{3}\sim 2.4 \pm 0.1$ ($\kappa _{0}\sim 0.9$)^43^, where the polytropic index is on the order $\gamma _{e}\sim 0.47$; or the kappa index in the fast solar wind $\mathrm {He^{+}}$ plasmas has reported to be on the order $\kappa _{3}\sim 2.65 \pm 0.27$ ($\kappa _{0}\sim 1.15$)^44^, where the polytropic index is on the order $\gamma _{e}\sim 0.53$. In these plasmas, the thermodynamics processes are very close to the escape state, where the transitions between the near/far-equilibrium states may be happen.

Furthermore, the hotter and denser space plasmas residing close to the thermal equilibrium, e.g., the kappa index in the lower solar corona $\mathrm {e^{-}}$ has reported to be on the order $\kappa _{3}\sim 17 \pm 7$ ($\kappa _{0}\sim 15.5$)^45^, where the polytropic index is on the order $\gamma _{e}\sim 0.94$; or the kappa index in the HII $\mathrm {e^{-}}$ regions has reported to be on the order $\kappa _{3}\sim 12 \pm 7$ ($\kappa _{0}\sim 10.5$)^46^, where the polytropic index is on the order $\gamma _{e}\sim 0.91$; or even the kappa index at the planetary nebulae has reported to be on the order $\kappa _{3}\sim 100 \pm 50$ ($\kappa _{0}\sim 100$)^47^, where we have the polytropic index very close to $\gamma _{e}\sim 1$. These are very close to the thermal equilibrium, where the related thermodynamics processes are isothermal.

Panel (b) in Fig. 1 shows the effect of the fractional ion to electron temperature on dispersion relation, where it has plotted for the fixed parameters $\kappa _{0}=2$ and $\gamma _{i}=3$, and for the typical values of the fractional ion to electron temperatures as $\sigma _{ie}=0$ (cold plasma), $\sigma _{ie}=0.1$, and $\sigma _{ie}=0.2$. It shows that the phase speed of IAWs increases with the temperature of the plasma ions, as is expected from the thermal pressure of the ions in the propagation of IAWs. Note that in our numerical analysis, we have considered the finite temperatures for the ions such as $\sigma _{ie}= T_{\infty ,i}/T_{\infty ,e}=0,0.01,0.1,0.2$.

We have to note that the Debye length in the typical space plasmas varies in different ranges, e.g., at the order $\lambda _{D,e}\sim 10^{-3}$ m in the earth ionospheric plasmas; at the order $\lambda _{D,e}\sim 10$ m in the solar wind plasma and the interstellar medium; at the order $\lambda _{D,e}\sim 10^{2}$ m in the magnetosphere plasmas; to the order $\lambda _{D,e}\sim 10^{5}$ m in the intergalactic medium^48^.

### Landau damping

Here, we may discuss the Landau damping of IAWs in space plasmas, where its formalism may be derived in terms of the invariant spectral index $\kappa _{0}$. Note that the formulation of the Landau damping may only be derived using the kinetic model equations (here, the Vlasov-Poisson’s equations), nor by using the hydrodynamics formalism. The main picture is the effective interaction/resonance of the IAWs and the plasma particles, where the ions may be accelerated by the wave and then the amplitude of the wave is decreased by losing its energy. This is the physical mechanism of Landau damping^49^. Here, the problem is understanding the effect of the supra-thermal particles in different thermodynamic states related to the typical space plasmas.

The imaginary part of the dielectric function of the IAWs reads as

15 $$\begin{aligned} D_{i}(k,\omega _{r})= - \frac{\pi }{k^{2}} \left[ \omega _{pi}^{2} \frac{\partial P_{i0}}{\partial u_{x}}+ \omega _{pe}^{2} \frac{\partial P_{e0}}{\partial u_{x}} \right] _{u_{x}=\frac{\omega _{r}}{|k|}}, \end{aligned}$$

then, by calculating the expressions $\frac{\partial P_{i(e)0}}{\partial u_{x}}|_{u_{x}=\frac{\omega _{r}}{|k|}}$ for the electrons (ions) and considering the solution of $\omega _{r}$ as given in Eq. (10), we may find the complete solution of $D_{i}(k,\omega _{r})$ for the invariant IAWs as follows

16 $$\begin{aligned} \begin{aligned} D_{i}(k,\omega _{r})&= -\sqrt{\frac{\pi }{2}} \frac{\omega _{r}}{|k^{3}|} \cdot \frac{\Gamma (\kappa _{0}+\frac{5}{2})}{\kappa _{0}^{5/2}\Gamma (\kappa _{0})} \\&\quad \times \Bigg \{ \omega _{pi}^{2} \left( \frac{m_{i}}{k_{B}T_{i}}\right) ^{\frac{3}{2}} \left[ 1+\frac{1}{\kappa _{0}} \cdot \left( \frac{\frac{1}{2}\frac{T_{e}}{T_{i}}}{(k\lambda _{De})^{2}+\frac{\kappa _{0}+1}{\kappa _{0}}}+\frac{3}{2} \right) \right] ^{-\kappa _{0}-\frac{5}{2}}\\&\quad + \omega _{pe}^{2} \left( \frac{m_{e}}{k_{B}T_{e}}\right) ^{\frac{3}{2}} \left[ 1+\frac{1}{\kappa _{0}} \cdot \left( \frac{\frac{1}{2}\frac{m_{e}}{m_{i}}}{(k\lambda _{De})^{2}+\frac{\kappa _{0}+1}{\kappa _{0}}}+\frac{3}{2} \frac{m_{e}}{m_{i}} \cdot \frac{T_{i}}{T_{e}} \right) \right] ^{-\kappa _{0}-\frac{5}{2}} \Bigg \} . \end{aligned} \end{aligned}$$

The imaginary part of the plasma normal modes has been derived by solving the relation $\omega _{i}=-\frac{D_{i}(k,\omega _{r})}{{\partial D_{r}(k,\omega _{r})}/{\partial \omega _{r}}}$^1^. In the plasma with finite ion temperature, we may find the damping rate of the invariant IAWs for the long wavelength modes ($k\lambda _{De}<1$) as follows

17 $$\begin{aligned} \begin{aligned} \omega _{i}&= -\sqrt{\frac{\pi }{8}} \; |\omega _{r}| \left[ \frac{1}{(k\lambda _{De})^{2}+\frac{\kappa _{0}+1}{\kappa _{0}}}+3\frac{T_{i}}{T_{e}} \right] ^{\frac{3}{2}} \cdot \frac{\Gamma (\kappa _{0}+\frac{5}{2})}{\kappa _{0}^{5/2}\Gamma (\kappa _{0})} \\&\quad \times \Bigg \{ \left( \frac{T_{e}}{T_{i}}\right) ^{\frac{3}{2}} \left[ 1+\frac{1}{\kappa _{0}} \cdot \left( \frac{\frac{1}{2}\frac{T_{e}}{T_{i}}}{(k\lambda _{De})^{2}+\frac{\kappa _{0}+1}{\kappa _{0}}}+\frac{3}{2} \right) \right] ^{-\kappa _{0}-\frac{5}{2}}\\&\quad + \left( \frac{m_{e}}{m_{i}}\right) ^{\frac{1}{2}} \left[ 1+\frac{1}{\kappa _{0}} \cdot \left( \frac{\frac{1}{2}\frac{m_{e}}{m_{i}}}{(k\lambda _{De})^{2}+\frac{\kappa _{0}+1}{\kappa _{0}}}+\frac{3}{2} \frac{m_{e}}{m_{i}} \cdot \frac{T_{i}}{T_{e}} \right) \right] ^{-\kappa _{0}-\frac{5}{2}} \Bigg \} . \end{aligned} \end{aligned}$$

We have to note that our solutions are valid for the longitudinal waves with the constraint of weak damping, i.e., $\omega _{i}\ll \omega _{r}$.

**Figure 2:**
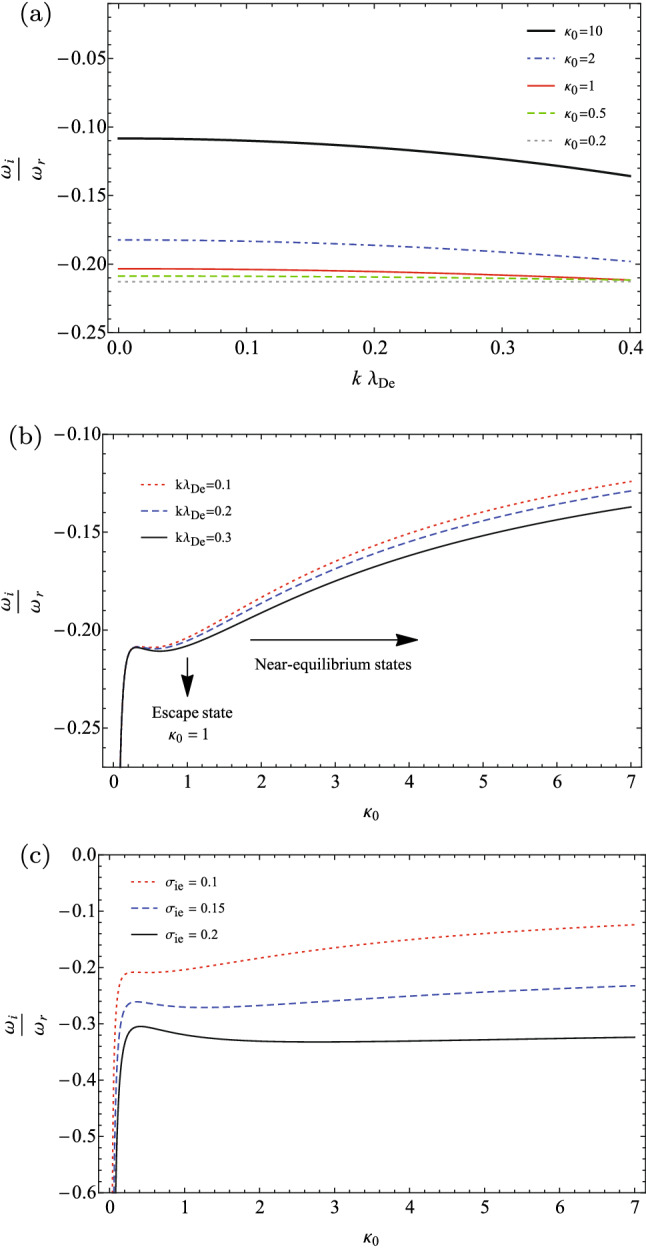
Damping rate of IAWs (**a**) with respect to the wave number for some typical invariant spectral indices when $\sigma _{ie}=0.1$; (**b**) with respect to the invariant spectral index for some typical wavelengths when $\sigma _{ie}=0.1$; (**c**) with respect to the invariant spectral index for some typical values of the fractional ion to electron temperature when $k\lambda _{De}=0.1$.

In Fig. 2, some features of the Landau damping of IAWs have numerically analyzed, where we have used the damping rate, as given in Eq. (17), normalized to the real part of the wave frequency. In panel (a), the normalized damping rate has plotted in terms of the wave number, for some typical invariant spectral indices and the fixed $\sigma _{ie}=0.1$. It shows that the (absolute value of) damping rate increases for the lower spectral indices, i.e., for the plasmas with more supra-thermal particles. So, the minimum damping of IAWs occurs in the equilibrium space plasmas, where the thermodynamic evolution of the system is isothermal ($\gamma _{e}\sim 1$). Furthermore, it is found that the effective resonance of the supra-thermal particles and the IAWs increases for the space plasmas closer to the anti-equilibrium stare, i.e., when the thermodynamic processes are sub-isothermal ($\gamma _{e}<1$). We have to note that our detailed comments in the prior subsection on different types of thermodynamic evolutions hold here, where it depends on the values of the spectral indices $\kappa _{0}$ and $\gamma _{e}$ in typical space plasma, as we have retained the same values as discussed in Fig. 1.

In panel (b) of Fig. 2, the variation of the damping rate with respect to the invariant spectral index has depicted for three typical wave numbers ($k\lambda _{De}=0.1,0.2,0.3$) and for a fixed fractional temperature as $\sigma _{ie}=0.1$. It shows that the damping rate of IAWs decreases for the longer wavelength modes. Furthermore, it tends to the minimum damping at the limit of $\kappa _{0}\rightarrow \infty$ (isothermal evolution of the plasma processes). The panel (c) of Fig. 2 shows the effect of fractional ion to electron temperature on the damping rate of IAWs, where it has been plotted for the fixed wave number $k\lambda _{De}=0.1$ and three typical fractional temperatures as $\sigma _{ie}=0.1$ , $\sigma _{ie}=0.15$, and $\sigma _{ie}=0.2$. It shows that the damping rate increases with the temperature of the ions in the plasma, and so the warmer space plasmas exhibit more effective Landau damping.

Interestingly, two panels (b) and (c) of Fig. 2 show that a critical spectral index exists in the vicinity $\kappa _{0}\sim 1$, in which the behavior of the IAWs has been distinguished for the lower/higher values of $\kappa _{0}\sim 1$. As we noted earlier, the stationary state with $\kappa _{0}=1$ corresponds to the escape state of the plasma, where the system can escape from the far-equilibrium regions ($\kappa _{0}<1$) towards the near-equilibrium regions ($\kappa _{0}>1$)^28^, passing from the state with the critical polytropic index as $\gamma _{e}\sim 0.5$.

**Figure 3:**
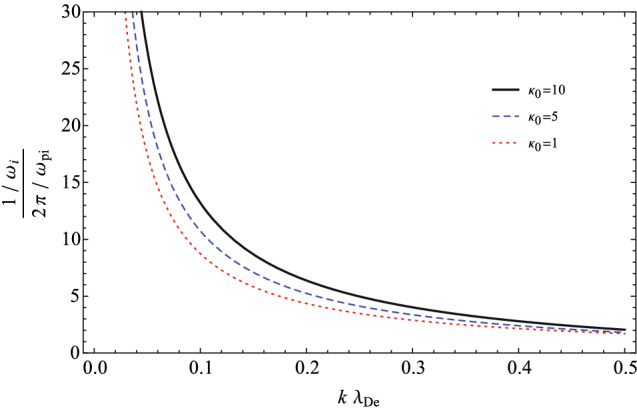
Landau damping time of IAWs with respect to the wave number for three typical invariant spectral indices when $\sigma _{ie}=0.1$.

In Fig. 3, we have analyzed the Landau damping time (in the period of the ion plasma oscillation), i.e., ($\frac{1/\omega _{i}}{2 \pi / \omega _{pi}}$), in terms of the wave number for three typical invariant spectral indices as $\kappa _{0}=1,5,10$ and the fixed fractional temperature as $\sigma _{ie}=0.1$. It shows that at a fixed wavelength, the damping time decreases for the plasmas with more supra-thermal particles (for the lower kappa indices). This confirms again the more prominent resonance of the supra-thermal particles and the IAWs in the space plasmas near the anti-equilibrium state. Moreover, the damping time of IAWs is considerable for the long wavelength modes, i.e., longer IAWs may survive more time in space.

For comparing our solutions here with the ones given in the classical plasmas, by considering $\frac{m_{e}}{m_{i}}\ll 1$ and $\frac{T_{i}}{T_{e}}\ll 1$, we may find the following estimation for the damping rate of IAWs in an ordinary electron-ion plasma as

18 $$\begin{aligned} \begin{aligned} \omega _{i}&= -\sqrt{\frac{\pi }{8}} \; \frac{|\omega _{r}|}{\left[ (k\lambda _{De})^{2}+\frac{\kappa _{0}+1}{\kappa _{0}}\right] ^{\frac{3}{2}}} \cdot \frac{\Gamma (\kappa _{0}+\frac{5}{2})}{\kappa _{0}^{5/2}\Gamma (\kappa _{0})} \\&\quad \times \Bigg \{ \left( \frac{T_{e}}{T_{i}}\right) ^{\frac{3}{2}} \left[ 1+\frac{1}{\kappa _{0}} \cdot \left( \frac{\frac{1}{2}\frac{T_{e}}{T_{i}}}{(k\lambda _{De})^{2}+\frac{\kappa _{0}+1}{\kappa _{0}}}+\frac{3}{2} \right) \right] ^{-\kappa _{0}-\frac{5}{2}} + \; \sqrt{\frac{m_{e}}{m_{i}}} \Bigg \} . \end{aligned} \end{aligned}$$

Furthermore, at the asymptotic limit $\kappa _{0}\rightarrow \infty$, our solutions are reduced to the classical IAWs solutions in a Maxwellian distributed electron-ion plasma, as follows

19a $$\begin{aligned} \frac{\omega _{r}^{2}}{k^{2}} & =   \frac{1}{k^2 \lambda _{De}^{2}+1} \cdot \frac{k_{B}T_{e}}{m_{i}} + \frac{3k_{B}T_{i}}{m_{i}} , \end{aligned}$$

19b $$\begin{aligned} \frac{\omega _{i}}{|\omega _{r}|} & =  - \; \sqrt{\frac{\pi }{8}} \left( \frac{1}{k^2 \lambda _{De}^{2}+1}+3\frac{T_{i}}{T_{e}} \right) ^{\frac{3}{2}} \nonumber \\ & \quad \times \Bigg [ \left( \frac{T_{e}}{T_{i}}\right) ^{\frac{3}{2}} \exp \left( - \frac{T_{e}/2T_{i}}{k^2 \lambda _{De}^{2}+1}-\frac{3}{2} \right) \;+ \sqrt{\frac{m_{e}}{m_{i}}} \Bigg ] , \end{aligned}$$

which are in agreement with the classical solutions as addressed in Ref.^17^. It is reminded that in many textbooks and literature^1–7^, by neglecting the ion temperature in comparison with the electron temperature, i.e., when $T_{i}\ll T_{e}$, the classical formulations of the IAWs have been summarized as

20a $$\begin{aligned} \omega _{r} & =  \frac{k \, c_{s}}{\sqrt{k^2 \lambda _{De}^{2}+1}}, \end{aligned}$$

20b $$\begin{aligned} \omega _{i} & =  - \frac{|\omega _{r}|\sqrt{\pi /8}}{(k^2 \lambda _{De}^{2}+1)^{\frac{3}{2}}} \Bigg [ \left( \frac{T_{e}}{T_{i}}\right) ^{\frac{3}{2}} \exp \left( - \frac{T_{e}/2T_{i}}{k^2 \lambda _{De}^{2}+1} \right) \;+ \sqrt{\frac{m_{e}}{m_{i}}} \Bigg ] , \end{aligned}$$

where $c_{s}=(\frac{k_{B}T_{e}}{m_{i}})^{\frac{1}{2}}$ is the effective ion-sound speed of the isothermal plasma, i.e. when $\gamma _{e}=1$.

### The perturbation expansion

In this section, we discuss the linear/nonlinear characteristics of the invariant IAWs and also the solitary wave solutions of a generalized Korteweg-de Vries (KdV) equation. It is emphasized that the KdV equation is a celebrated integrable equation in the nonlinear physics^50^ which describes the long waves in a dispersive media such as the plasma^51^.

By considering the potential energy of the electrons as $\Phi _{e}=-e\phi (x)$ in the canonical distribution function, where $\phi (x)$ is the electrostatic potential of the ion waves, one may find the number density of the invariant kappa distributed electrons by calculating the statistical moments of the canonical distribution over the velocity as follows^52^

21 $$\begin{aligned} n_{e}(x)=n_{\infty ,e} \cdot \left[ 1-\frac{1-\gamma _{e}}{\gamma _{e}}\cdot \frac{e\phi (x)}{k_{B}T_{\infty ,e}}\right] ^{\frac{1}{\gamma _{e}-1}}, \end{aligned}$$

where $n_{\infty ,e}$ and $T_{\infty ,e}$ are respectively the number density and the temperature of the electrons at zero potential; and $\gamma _{e}$ is the generalized polytropic index of kappa distributed electrons which is given by the formula $\gamma _{e}=\frac{\kappa _{0}+\frac{1}{2}d_{\Phi ,e}}{\kappa _{0}+1+\frac{1}{2}d_{\Phi ,e}}$. Here $d_{\Phi ,e}$ is the potential degrees of freedom for the electrons in the presence of the ion waves’ potential and it is given by $\frac{1}{2}d_{\Phi ,e}= -\frac{e\langle \phi (x)\rangle }{k_{B}T_{\infty ,e}}$. It is emphasized that if $d_{\Phi ,e}$ is positive, then $\gamma _{e}$ is less than one, and if it is negative, then $\gamma _{e}$ can be either larger or smaller than one^53^. Noting that the ion waves’ potential (with respect to the potential at infinity) is positive, $\phi >0$, so $d_{\Phi ,e}$ is negative and then $\gamma _{e}$ may be either larger or smaller than one.

By using the generalized formulation of the ion-sound speed and Debye length, we use a set of well-defined normalized parameters as follows

22 $$\begin{aligned} \frac{x}{\lambda _{D,\gamma _{e}}}\rightarrow x^{'}, \; \; \; \frac{t}{\omega _{pi}^{-1}}\rightarrow t^{'}, \; \; \; \frac{v_{i}}{c_{s,\gamma _{e}}}\rightarrow v^{'}, \; \; \; \frac{n_{i}}{n_{\infty ,i}}\rightarrow n^{'}, \; \; \; \frac{p_{i}}{n_{\infty ,i}k_{B}T_{\infty ,i}}\rightarrow p^{'}, \; \; \; \frac{e\phi }{k_{B}T_{\infty ,e}}\rightarrow \phi ^{'}, \; \; \; \end{aligned}$$

where, $\lambda _{D,\gamma _{e}}=\sqrt{\gamma _{e}\frac{\varepsilon _{0}k_{B}T_{\infty ,e}}{e^{2}n_{\infty ,e}}}$ is the generalized Debye length via the kappa distributed electrons^34^, $\omega _{pi}=\sqrt{\frac{Z_{i}^{2}e^{2}n_{\infty ,i}}{\varepsilon _{0}m_{i}}}$ is the ion oscillation frequency, and $c_{s,\gamma _{e}}=\sqrt{\gamma _{e}\frac{Z_{i}k_{B}T_{\infty ,e}}{m_{i}}}$ is the generalized ion-sound speed of the plasma by the kappa distributed electrons^36^. Furthermore, $n_{\infty ,i}$ is the number density of the ions at infinity, which satisfies the quasi-neutrality conditions of the plasma as $Z_{i}n_{\infty ,i}=n_{\infty ,e}$. There exist a simple relation between the ion oscillation frequency, the generalized Debye length, and the generalized ion-sound speed as $\omega _{pi}\cdot \lambda _{D,\gamma _{e}}=c_{s,\gamma _{e}}$^36^. Furthermore, in the asymptotic limit $\kappa _{0}\rightarrow \infty$ or $\gamma _{e}\rightarrow 1$ (Maxwellian plasma), the classical relation $\omega _{pi}\cdot \lambda _{D,\infty }=c_{s,\infty }$ has been retained between the classical parameters, where the $\lambda _{D,\infty }=\sqrt{\frac{\varepsilon _{0}k_{B}T_{\infty ,e}}{e^{2}n_{\infty ,e}}}$ and $c_{s,\infty }=\sqrt{\frac{Z_{i}k_{B}T_{\infty ,e}}{m_{i}}}$ are the classical Debye length and the ion-sound speed, respectively. Then, the normalized equations for propagation of the invariant IAWS may be written as

23a $$\begin{aligned}{} & {} \frac{\partial n^{'}}{\partial t^{'}} + \frac{\partial (n^{'}v^{'}) }{\partial x^{'}}=0, \end{aligned}$$

23b $$\begin{aligned}{} & {} \frac{\partial v^{'}}{\partial t^{'}} + v^{'} \frac{\partial v^{'}}{\partial x^{'}} = -\frac{1}{\gamma _{e}} \frac{\partial \phi ^{'}}{\partial x^{'}} - \frac{\sigma _{ie}}{ Z_{i} \gamma _{e}} \frac{1}{n^{'}}\frac{\partial p^{'}}{\partial x^{'}}, \end{aligned}$$

23c $$\begin{aligned}{} & {} \frac{\partial p^{'}}{\partial t^{'}} + v^{'} \frac{\partial p^{'}}{\partial x^{'}} + \gamma _{i}\; p^{'} \frac{\partial v^{'}}{\partial x^{'}}=0, \end{aligned}$$

23d $$\begin{aligned}{} & {} \frac{\partial ^{2}\phi ^{'}}{\partial {x^{'}}^{2}}= \gamma _{e} \left[ \left( 1-\frac{1-\gamma _{e}}{\gamma _{e}} \phi ^{'} \right) ^{\frac{1}{\gamma _{e}-1}}-n^{'} \right] . \end{aligned}$$

For deriving the KdV equation and its solitary wave solutions, we use the stretched space and time coordinates $\xi$ and $\tau$ as follows^51,54–58^

24a $$\begin{aligned} \xi & =  \delta ^{\frac{1}{2}}(x^{'}-\lambda ^{'} \, t^{'}), \end{aligned}$$

24b $$\begin{aligned} \tau & =  \delta ^{\frac{3}{2}} t^{'}, \end{aligned}$$

where $\lambda ^{'}$ is the extended phase velocity of the ion waves in terms of the generalized ion-sound speed as defined in our normalization, i.e., $\lambda ^{'}=\frac{V_{phase}}{c_{s,\gamma _{e}}}$, and $\delta$ is a small parameter for expanding the physical parameters about the equilibrium values as follows

25a $$\begin{aligned} n^{'}(\xi ,\tau ) & =  1+\delta n_{1}(\xi ,\tau )+\delta ^{2} n_{2}(\xi ,\tau )+\cdots \end{aligned}$$

25b $$\begin{aligned} v^{'}(\xi ,\tau ) & =  \delta v_{1}(\xi ,\tau )+\delta ^{2} v_{2}(\xi ,\tau )+\cdots \end{aligned}$$

25c $$\begin{aligned} p^{'}(\xi ,\tau ) & =  1+\delta p_{1}(\xi ,\tau )+\delta ^{2} p_{2}(\xi ,\tau )+\cdots \end{aligned}$$

25d $$\begin{aligned} \phi ^{'}(\xi ,\tau ) &  = \delta \phi _{1}(\xi ,\tau )+\delta ^{2} \phi _{2}(\xi ,\tau )+\cdots \end{aligned}$$

#### The lowest order of perturbation: the phase speed

By considering the boundary conditions as $n,p\rightarrow 1$ and $v,\phi \rightarrow 0$ when $|\xi |\rightarrow \infty$ for having a localized solitary wave, integrating the normalized Eq. (23) in the lowest order of the perturbation (the order $\delta ^{3/2}$ in the continuity equation, the momentum transfer equation, and the pressure evolution equation, and the order $\delta ^{1}$ in the Poisson equation), we have the following equations

26a $$\begin{aligned} \lambda ^{'} n_{1} & =  v_{1}, \end{aligned}$$

26b $$\begin{aligned} \lambda ^{'} v_{1} & =  \frac{\phi _{1}}{\gamma _{e}}+\frac{\sigma _{ie}}{Z_{i}\gamma _{e}} p_{1}, \end{aligned}$$

26c $$\begin{aligned} \lambda ^{'} p_{1} & =  \gamma _{i} v_{1}, \end{aligned}$$

26d $$\begin{aligned} \phi _{1} & =  \gamma _{e} n_{1}, \end{aligned}$$

where the solution leads to the extended phase speed of the IAWs as follows

27 $$\begin{aligned} \lambda ^{'}=\sqrt{1+\frac{\gamma _{i}\sigma _{ie}}{Z_{i}\gamma _{e}}}. \end{aligned}$$

Noting that $c_{s,\gamma _{e}}=\sqrt{\gamma _{e}}c_{s,\infty }$, we may also find an explicit expression for the normalized phase speed of the IAWs with respect to the isothermal ion-sound speed, i.e., $\lambda _{\infty }=\frac{V_{phase}}{c_{s,\infty }}$ as follows

28 $$\begin{aligned} \lambda _{\infty }=\sqrt{\gamma _{e}+\frac{\gamma _{i}\sigma _{ie}}{Z_{i}}}. \end{aligned}$$

We see that the (extended/isothermal) phase speed is a function of the generalized adiabatic index $\gamma _{e}$ and so it is a sensitive function of the stationary state of the plasma. This confirms that the ion-sound speed varies in different thermodynamic states of the plasmas, in agreement with the results of Ref.^36^.

Nothing to our extended normalization process, we may find that the isothermal formulation of the phase speed as given in Eq. (28) may describe the pure effect of the adiabatic index $\gamma _{e}$ on the phase speed of the IAWs without any redundancy.

**Figure 4:**
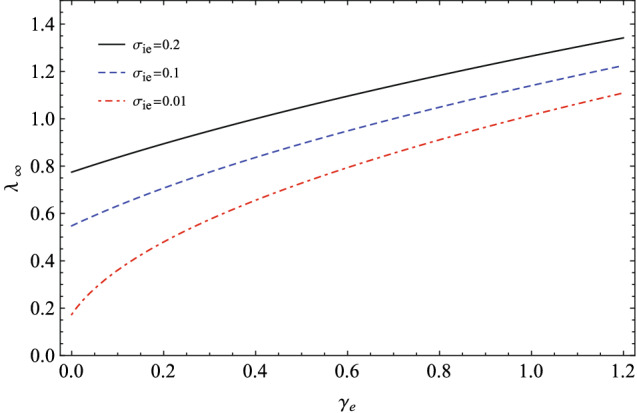
The variation of the isothermal phase speed of IAWs with respect to the generalized adiabatic index $\gamma _{e}$ for some fractional ion to electron temperatures when $Z_{i}=1$ and $\gamma _{i}=3$.

In Fig. 4, we have depicted the variation of the isothermal phase speed $\lambda _{\infty }$ with respect to the generalized adiabatic index $\gamma _{e}$ for some fractional ion to electron temperatures, when the polytropic index of the ions is $\gamma _{i}=3$ (corresponding to the one-dimensional compression/rarefaction of the ion waves). It shows that the phase speed of the IAWs increases with $\gamma _{e}$ towards its maximum values in the vicinity of the isothermal states ($\gamma _{e}\approx 1$). This may be explained by the fact that the ion-sound speed takes its maximum value for an equilibrium Maxwellian plasma^36^.

We remember that we may have $\gamma _{e}$ either larger or smaller than one, because of the perturbations of the potential in the ion waves. As we noted earlier, here we have the positive potentials ($\phi >0$) and the potential degrees of freedom for the electrons with negative values ($d_{\Phi ,e}<0$). So, we have considered the super-isothermal stationary states with some adiabatic indices larger than one in our numerical analysis. Furthermore, Fig. 4 shows that the phase speed of the IAWs increases with the temperature of the plasma ions.

For comparing our results here with the previous ones, we have expanded the isothermal phase speed of the IAWs in the limit $T_{i} \ll T_{e}$ ($\sigma _{ie} \ll 1$) as

29 $$\begin{aligned} \lambda _{\infty } \simeq \gamma _{e}+ \frac{\gamma _{i}\sigma _{ie}}{2Z_{i}}, \end{aligned}$$

where it takes its maximum value in the case of an isothermal plasma (by considering $\gamma _{e}\rightarrow 1$) and when the polytropic index of the ions is $\gamma _{i}=3$ (the one-dimensional propagation of the IAWs), as $\lambda _{\infty } \simeq 1+ \frac{3\sigma _{ie}}{2Z_{i}}$. This is in agreement with the reported results of Refs.^54,59^, and also it confirms that the small amplitude ion-acoustic solitons in a Maxwellian plasma may propagate with the speeds around 1 Mach^50^.

A similar method for deriving the phase speed of the IAWs and the ion-sound speed in the context of the hydrodynamics formalism is referring to the linear dispersion relation. We assume that the perturbed variables oscillate as $exp[i(\vec {k^{'}}\cdot \vec {R}-\omega ^{'} t)]$, where $\vec {k^{'}}$ and $\omega ^{'}$ are the normalized wave vector and wave frequency, respectively, and $\vec {R}$ is the position vector. Then by simultaneously solving the Eqs. (23) and neglecting the terms of the second and higher orders, we may find a generalized linear dispersion relation for the IAWs as follows

30 $$\begin{aligned} \frac{{\omega ^{'}}^{2}}{{k^{'}}^{2}}=\frac{1}{1+{k^{'}}^{2}}+\frac{\gamma _{i}\sigma _{ie}}{Z_{i}\gamma _{e}}. \end{aligned}$$

Then, the extended phase speed of the IAWs may be re-derived by using the formula $\lambda ^{'}=\lim _{k^{'}\rightarrow 0}\frac{\omega ^{'}}{k^{'}}$ as that given in Eq. (27).

It is informative to come back to the non-normalized variables by using the transformations as

31 $$\begin{aligned} \omega ^{'} \rightarrow \frac{\omega }{\omega _{pi}}, \; \; \; \; \; \; k^{'} \rightarrow k \lambda _{D,\gamma _{e}}. \end{aligned}$$

Then, the linear dispersion relation takes the following form

32 $$\begin{aligned} \frac{{\omega }^{2}}{{k}^{2}}=\frac{1}{1+(k \lambda _{D,\gamma _{e}})^{2}}\frac{\gamma _{e}Z_{i}k_{B}T_{\infty ,e}}{m_{i}}+\frac{\gamma _{i}k_{B}T_{i}}{m_{i}}. \end{aligned}$$

By this formalism, we may re-derive the explicit formulation of the generalized ion-sound speed as $c_{s}(\gamma _{e},\gamma _{i})=\left( \frac{\gamma _{e}Z_{i}k_{B}T_{e}}{m_{i}} +\frac{\gamma _{i}k_{B}T_{i}}{m_{i}} \right) ^{\frac{1}{2}}$, where $\gamma _{e}=\frac{\kappa _{0}+\frac{1}{2}d_{\Phi ,e}}{\kappa _{0}+1+\frac{1}{2}d_{\Phi ,e}}$ and $\gamma _{i}=\frac{d_{i}+2}{d_{i}}$, which is in agreement with the results given in Ref.^36^ and also in agreement with the Eq. (13) in the previous section, where a kinetic approach had been used in the special case when $Z_{i}=1$ and $d_{i}=1$ (the one-dimensional propagation of the IAWs).

For avoiding the misunderstanding, we have to note that the linearized dispersion relation given in Eq. (30) is similar to the one as given in Eq. (14), with a minor difference in defining the normalized wave number $k^{'}$, where $k^{'}$ in Eq. (30) is an extended parameter as defined in Eq. (31). By defining the relevant normalized wave number as ${k^{'}}_{\infty } \rightarrow k \lambda _{D,\infty }$, the Eq. (30) may be transformed as

33 $$\begin{aligned} \frac{\omega ^{'}}{{k^{'}}_{\infty }}= \left( \frac{1}{{k^{'}}_{\infty }^{2}+\frac{1}{\gamma _{e}}}+\frac{\gamma _{i}\sigma _{ie}}{Z_{i}}\right) ^{\frac{1}{2}}, \end{aligned}$$

which is quite identical to the Eq. (14) when $Z_{i}=1$ and $d_{\Phi ,e}=0$.

**Figure 5:**
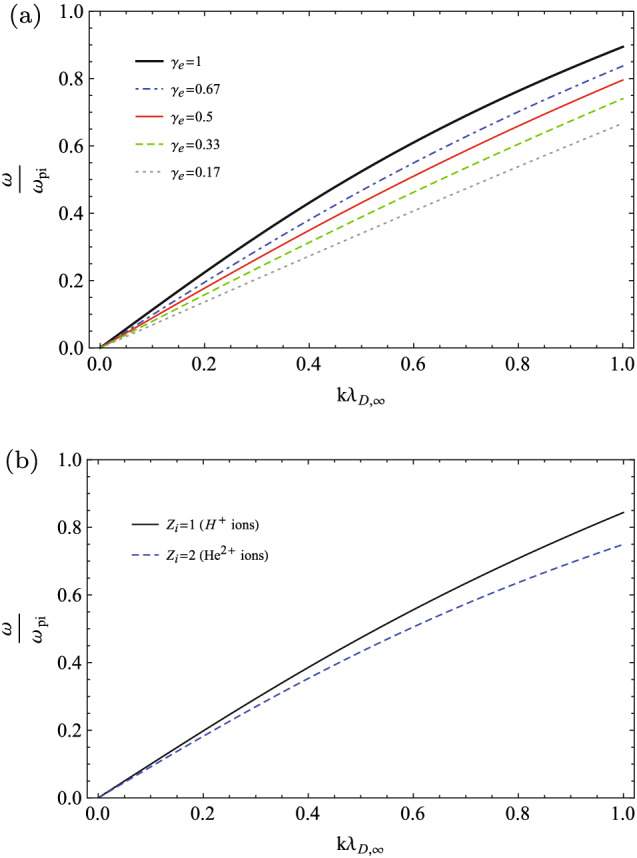
The linear dispersion relation diagram: (**a**) for some adiabatic indices in accordance with the data in Fig. 1a when $\sigma _{ie}=0.1$, $\gamma _{i}=3$ and $Z_{i}=1$; (**b**) for the Hydrogen and Helium plasmas.

In panel (a) of Fig. 5, we have depicted the variations of the linear dispersion relation as given in Eq. (33) for some adiabatic indices in accordance with the data on panel (a) in Fig. 1 when $\sigma _{ie}=0.1$, $\gamma _{i}=3$ and $Z_{i}=1$. As is expected the results of the hydrodynamics approach are in agreement with the ones in the kinetic theory formalism. Furthermore, the panel (b) of Fig. 5 is for the sake of comparison of the dispersion relation diagrams in two cases, i.e., the plasmas with Hydrogen ions ($Z_{i}=1$) and Helium ions ($Z_{i}=2$) when $\sigma _{ie}=0.1$, $\gamma _{e}=0.7$ and $\gamma _{i}=3$. As it is expected, it shows that the phase speed is slower for the plasmas with heavier ions.

#### The second order of perturbation: the KdV equation

The normalized Eq. (23) in the next order of the perturbation (the order $\delta ^{5/2}$ in the continuity equation, the momentum transfer equation, and the pressure evolution equation, and the order $\delta ^{2}$ in the Poisson equation) may result in the following set of partial differential equations

34a $$\begin{aligned}{} & {} \frac{\partial n_{1}}{\partial \tau }- \lambda ^{'} \frac{\partial n_{2}}{\partial \xi } +\frac{\partial v_{2}}{\partial \xi } +2\lambda ^{'} n_{1} \frac{\partial n_{1}}{\partial \xi }=0, \end{aligned}$$

34b $$\begin{aligned}{} & {} \lambda ^{'} \frac{\partial n_{1}}{\partial \tau } -\lambda ^{'} \frac{\partial v_{2}}{\partial \xi }+ n_{1} \frac{\partial n_{1}}{\partial \xi } + \frac{1}{\gamma _{e}} \frac{\partial \phi _{2}}{\partial \xi } + \frac{\sigma _{ie}}{Z_{i}\gamma _{e}} \frac{\partial p_{2}}{\partial \xi }=0, \end{aligned}$$

34c $$\begin{aligned}{} & {} \gamma _{i} \frac{\partial n_{1}}{\partial \tau }-\lambda ^{'} \frac{\partial p_{2}}{\partial \xi } +\gamma _{i}(1+\gamma _{i})\lambda ^{'} n_{1} \frac{\partial n_{1}}{\partial \xi } + \gamma _{i} \frac{\partial v_{2}}{\partial \xi }=0, \end{aligned}$$

34d $$\begin{aligned}{} & {} \frac{\partial ^{2} n_{1}}{\partial \xi ^{2}}-\frac{\phi _{2}}{\gamma _{e}}-\frac{2-\gamma _{e}}{2} n_{1}^{2}+ n_{2}=0, \end{aligned}$$

where our solutions in the first order of the perturbation have been also used. By simultaneously solving these equations and also by using the resultant formula for the extended phase speed, i.e., Eq. (27), we may derive an evolution equation for propagation of the invariant IAWs as a generalized KdV equation as follows

35 $$\begin{aligned} \frac{\partial U}{\partial \tau } +\alpha (\gamma _{e},\gamma _{i}) \frac{\partial ^{3} U}{\partial \xi ^{3}} + \beta (\gamma _{e},\gamma _{i}) U \frac{\partial U}{\partial \xi }=0, \end{aligned}$$

where *U* stands for the first-order perturbed variables, i.e., $n_{1},v_{1},p_{1},\phi _{1}$. Here, $\alpha (\gamma _{e},\gamma _{i})$ is the generalized dispersion coefficient that describes the spreading of the wave packet because of the phase relations between different components of the wave, and $\beta (\gamma _{e},\gamma _{i})$ is the generalized nonlinear coefficient that is related to the steepening of the wave when it propagates in the plasma, where its physical reason is that the higher amplitude components of the wave travel with more speeds in comparison with the lower amplitude ones. As we see, the generalized dispersion and nonlinear coefficients are the functions of $\gamma _{e}$ and $\gamma _{i}$, where they are as follows

36a $$\begin{aligned}{} & {} \alpha (\gamma _{e},\gamma _{i})=\frac{1}{2\sqrt{1+\frac{\gamma _{i}\sigma _{ie}}{Z_{i}\gamma _{e}}}}, \end{aligned}$$

36b $$\begin{aligned}{} & {} \beta (\gamma _{e},\gamma _{i})=\frac{1+\gamma _{e}+\frac{\gamma _{i}(1+\gamma _{i})\sigma _{ie}}{Z_{i}\gamma _{e}}}{2\sqrt{1+\frac{\gamma _{i}\sigma _{ie}}{Z_{i}\gamma _{e}}}}. \end{aligned}$$

These generalized formulas are valid for the *d*-dimensional IAWs, e.g., for the propagation of the IAWs in one dimension, where $d_{i}=1$ and the ions polytropic index is $\gamma _{i}=3$, or for the propagation of the IAWs in three dimensions, where $d_{i}=3$ and the ions polytropic index is $\gamma _{i}=\frac{5}{3}$.

**Figure 6:**
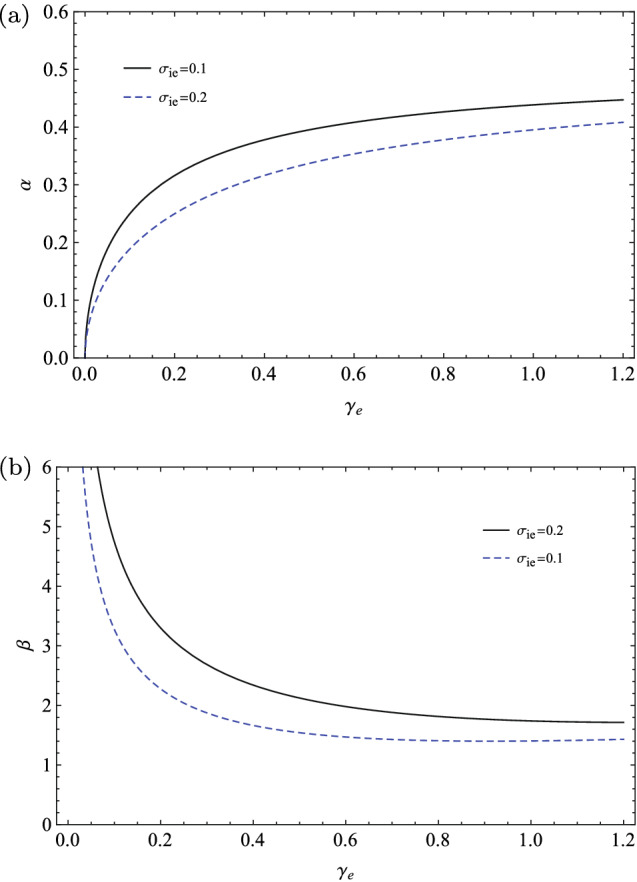
The variations of (**a**) the generalized dispersion coefficient and (**b**) the generalized nonlinear coefficient with respect to the extended adiabatic index $\gamma _{e}$ for two typical fractional temperatures of ions to electrons when $\gamma _{i}=3$ and $Z_{i}=1$.

In Fig. 6, we have numerically depicted the variations of the generalized dispersion coefficient (the panel(a)) and the generalized nonlinear coefficient (the panel(b)) in terms of the generalized adiabatic index $\gamma _{e}$ for two typical fractional temperatures when $\gamma _{i}=3$ and $Z_{i}=1$. As we see the dispersion coefficient increases with $\gamma _{e}$ but the nonlinear coefficient decreases with $\gamma _{e}$ and also both of them decrease with $\sigma _{ie}$. We emphasize that for having a stable solitary wave, the effects of the two terms, i.e., $\alpha (\gamma _{e},\gamma _{i})$ and $\beta (\gamma _{e},\gamma _{i})$ have balanced.

In the special case of the isothermal electron populations ($\gamma _{e}\rightarrow 1$) and one-dimensional evolution of the adiabatic ions ($\gamma _{i}=3$), our generalized dispersion and nonlinear coefficients are in agreement with the results given in Refs.^54,57^. Furthermore, in the limit of cold ions ($\sigma _{ie}\rightarrow 0$), the isothermal electrons ($\gamma _{e}\rightarrow 1$), and one-dimensional propagation of the ion wave ($\gamma _{i}=3$), our result in Eq. (35) may reduce to the standard KdV equation in an ordinary Maxwellian plasma as $\frac{\partial U}{\partial \tau } +\frac{1}{2} \frac{\partial ^{3} U}{\partial \xi ^{3}}+ U \frac{\partial U}{\partial \xi }=0$, where the dispersion coefficient is $\frac{1}{2}$ and the nonlinear coefficient is 1^7,50^. Note that at the cold plasma regime ($\sigma _{ie}\rightarrow 0$), the nonlinear coefficient is linearly proportional to $\gamma _{e}$ and it increases from $\beta (\gamma _{e}=0)=1/2$ to the asymptotic limit $\beta (\gamma _{e}=1)=1$ for a Maxwellian distributed plasma. At this regime, the dispersion coefficient tends smoothly to the asymptotic limit $\alpha \rightarrow 1/2$.

#### The solitary wave solutions

We may derive the solitary wave solutions of the Eq. (35) by considering the solutions in the co-moving frame of the wave. So by using the Galilean transformation as $\xi ^{'}=\xi -c\tau$, where *c* is the normalized wave speed and $\xi ^{'}$ is the positional variable of the soliton in the reference frame of the wave, we have the following transformed KdV equation in terms of the single variable $\xi ^{'}$ as

37 $$\begin{aligned} -c \frac{\textrm{d} U}{\textrm{d} \xi ^{'}} +\alpha (\gamma _{e},\gamma _{i}) \frac{\textrm{d}^{3} U}{\textrm{d} {\xi ^{'}}^{3}} + \beta (\gamma _{e},\gamma _{i}) U \frac{\textrm{d} U}{\textrm{d} \xi ^{'}}=0. \end{aligned}$$

By integrating the Eq. (37) and considering the boundary conditions for having a localized solitary pulse as $U(\xi ^{'}) , \frac{\textrm{d} U(\xi ^{'})}{\textrm{d} \xi ^{'}} , \frac{\textrm{d}^{2} U(\xi ^{'})}{\textrm{d} {\xi ^{'}}^{2}}\rightarrow 0$ when $|\xi ^{'}|\rightarrow \infty$, we may have the following differential equation

38 $$\begin{aligned} \left( \frac{\textrm{d} U(\xi ^{'})}{\textrm{d} \xi ^{'}}\right) ^{2}=\frac{\beta (\gamma _{e},\gamma _{i}){U(\xi ^{'})}^{2}}{3 \alpha (\gamma _{e},\gamma _{i})} \left( \frac{3c}{\beta (\gamma _{e},\gamma _{i})}-U(\xi ^{'})\right) . \end{aligned}$$

Then, by integrating the Eq. (38) and considering the boundary conditions, we have the solitary wave solution in terms of $\xi ^{'}$ as

39 $$\begin{aligned} U(\xi ^{'})=\frac{3c}{\beta (\gamma _{e},\gamma _{i})} sech^{2} \left( \sqrt{\frac{c}{4 \alpha (\gamma _{e},\gamma _{i})}} \xi ^{'} \right) , \end{aligned}$$

or in terms of the positional parameter $\xi$ in the original reference frame as

40 $$\begin{aligned} U(\xi -c\tau )=\textrm{U}_{max}(\gamma _{e},\gamma _{i}) sech^{2} \left[ \frac{(\xi -c\tau )}{\Delta (\gamma _{e},\gamma _{i})} \right] , \end{aligned}$$

where $\textrm{U}_{max}(\gamma _{e},\gamma _{i})$ and $\Delta (\gamma _{e},\gamma _{i})$ are respectively the maximum amplitude and the pulse width of the soliton. They have the following expressions in terms of the polytropic indices $\gamma _{e}$ and $\gamma _{i}$ as

41a $$\begin{aligned} \textrm{U}_{max}(\gamma _{e},\gamma _{i}) & =   \frac{6c\sqrt{1+\frac{\gamma _{i}\sigma _{ie}}{Z_{i}\gamma _{e}}}}{1+\gamma _{e}+\frac{\gamma _{i}(1+\gamma _{i})\sigma _{ie}}{Z_{i}\gamma _{e}}}, \end{aligned}$$

41b $$\begin{aligned} \Delta (\gamma _{e},\gamma _{i}) & =  \frac{\sqrt{2/c}}{\left( 1+\frac{\gamma _{i}\sigma _{ie}}{Z_{i}\gamma _{e}}\right) ^{1/4}}. \end{aligned}$$

**Figure 7:**
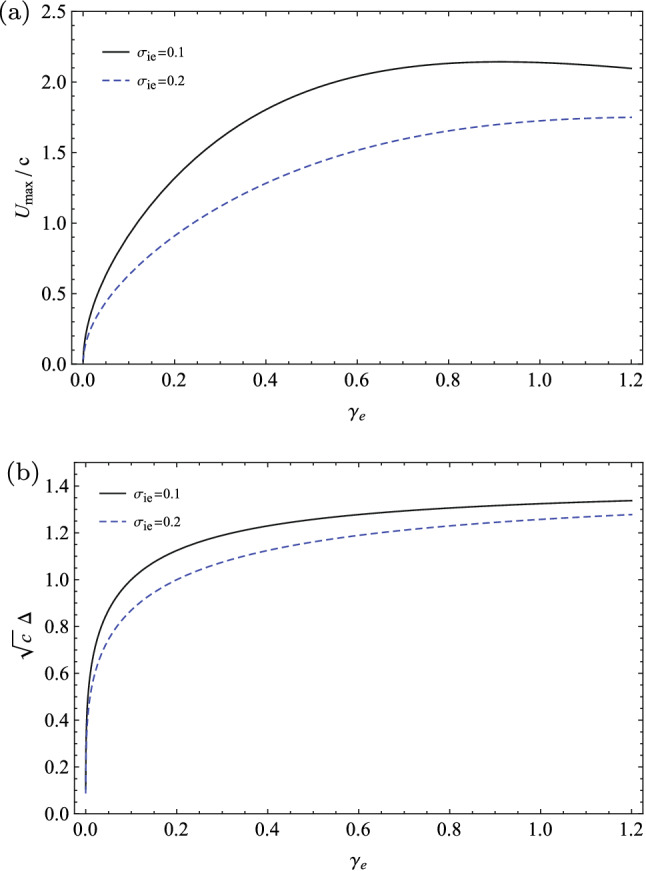
The variations of (**a**) the maximum amplitude and (**b**) the pulse width of the solitary waves with respect to the generalized adiabatic index $\gamma _{e}$ for two typical fractional temperatures of the ions to electrons when $\gamma _{i}=3$ and $Z_{i}=1$.

In two panels of Fig. 7, we have numerically plotted the variations of the maximum amplitude and the pulse width of the solitary wave solution in terms of the spectral index $\gamma _{e}$ for two typical fractional temperatures as $\sigma _{ie}=0.1,0.2$ when $\gamma _{i}=3$ and $Z_{i}=1$. Figure 7 shows that both the maximum amplitude and the pulse width of the soliton increase with $\gamma _{e}$ and they decrease with $\sigma _{ie}$. At the cold plasma regime ($\sigma _{ie}\rightarrow 0$), the maximum amplitude of the ion-acoustic soliton is given by $\textrm{U}_{max}(\gamma _{e})=\frac{6c}{1+\gamma _{e}}$, so it decreases from the asymptotic limit $\textrm{U}_{max}(\gamma _{e}\rightarrow 0)=6c$ to the minimum value $\textrm{U}_{max}(\gamma _{e}=1)=3c$ for a Maxwellian distributed plasma. At this regime, the variation of the soliton amplitude with respect to the extended adiabatic index $\gamma _{e}$ is opposite of the one for the warm ions. Furthermore, when $\sigma _{ie}\rightarrow 0$ is considered, the width of the soliton pulse is only dependent on the inverse of the square root of the soliton speed ($\Delta =\sqrt{\frac{2}{c}}$), as it is expected from the nonlinear classical KdV theory^50^.

**Figure 8:**
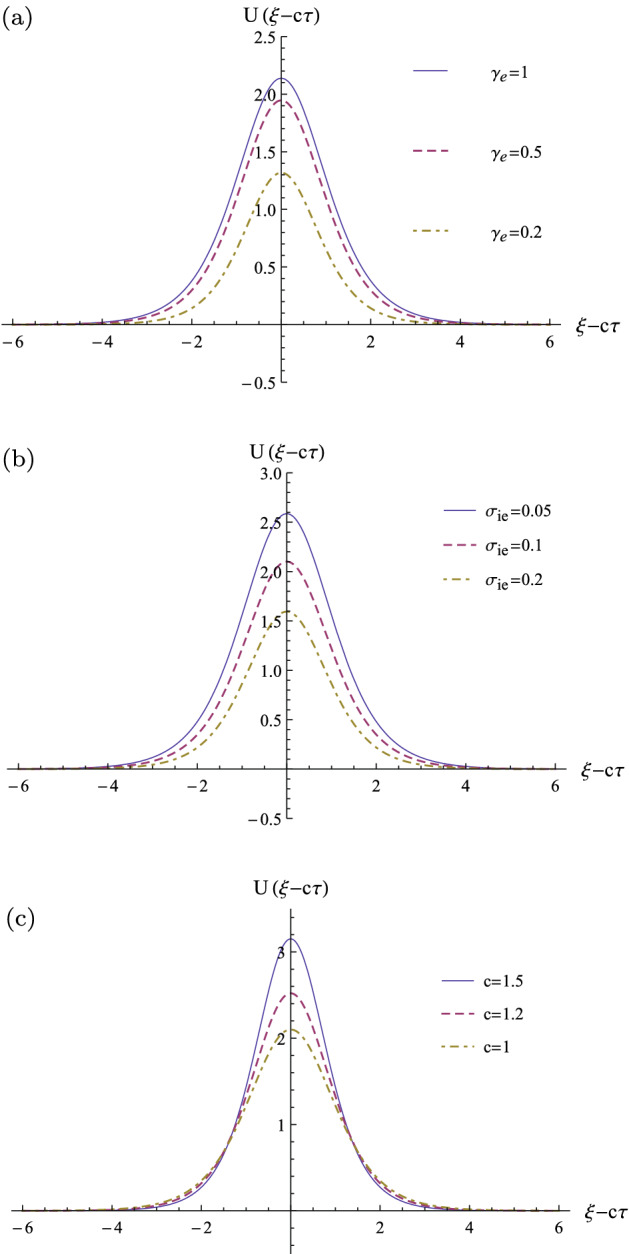
The profiles of the solitary wave pulses in the co-moving wave frame: (**a**) for some typical adiabatic indices when $c=1$ and $\sigma _{ie}=0.1$; (**b**) for some typical fractional ion to electron temperatures when $c=1$ and $\gamma _{e}=0.7$; (**c**) for some typical normalized soliton speeds when $\gamma _{e}=0.7$ and $\sigma _{ie}=0.1$. Here, it is assumed that $Z_{i}=1$ and $\gamma _{i}=3$ for all the profiles.

Finally, in three panels of Fig. 8, we have numerically depicted the two-dimensional profiles of the solitary wave pulses in the co-moving wave frame, where we have shown the effects of the adiabatic index variation (panel (a)); the fractional ion to electron temperature (panel (b)); and the soliton speed variation (panel (c)) on the profiles of the soliton pulses. Panel (a) shows that close to the equilibrium state ($\gamma _{e}\rightarrow 1$), the amplitude and the pulse width of the solitary wave increase. Panel (b) shows the amplitude and the width of the solitary wave pulse decrease related to the temperature of the ions. Panel (c) confirms the celebrated feature of the nonlinear wave propagation that more speed solitons have more amplitude and narrower pulse width^50^.

As a supplementary note, we have to emphasize that the positional parameters $x^{'}$ and $\xi$ and also the velocity parameters $v^{'}$ and *c* in our analysis of the solitary wave solutions are the extended parameters, where they have been defined in terms of the generalized Debye length and the generalized ion-sound speed, respectively, as given in Eqs. (22) and (24). So, we have to anticipate that by using the relevant non-extended parameters such as $\xi _{\infty }$ and $c_{\infty }$, where they may be defined in terms of the isothermal Debye length and ion-sound speed, the variation of the solitary wave as $U_{\infty } (\xi _{\infty }-c_{\infty }\tau )$ in terms of the generalized adiabatic index $\gamma _{e}$ may be different from our results here. To our knowledge, the best and more generalized expressions for the normalization of the parameters are the ones we have used in this study. Especially, the normalization of the wave speed in terms of the true ion-sound speed becomes very important for deriving the well-defined Mach number values in the propagation of the large amplitude solitons, shocks, and double layers in the plasma^60–62^ or analysis of the Mach number domains in the plasma sheaths^52^.

## Conclusion

In this paper, we discussed the linear and nonlinear features of the invariant IAWs in astrophysics and space plasmas. Our formulations were developed in the modern kappa distribution formalism in terms of an invariant kappa index, $\kappa _{0}$, as of zero dimensionality spectral index, and also in terms of the extended adiabatic indices of the plasma species, $\gamma _{j}$, where we discussed the pure effect of the thermodynamics evolutions on the propagation of the IAWs. The kinetic Vlasov-Poisson equations were used in the linear regime and the hydrodynamic fluid equations were used both in the linear and nonlinear regimes for the invariant ion waves. We derived the most generalized formulations of the dispersion relation, the ion-sound speed, the Landau damping, and the solitary wave solutions in an extended co-moving frame of the wave, in terms of $\kappa _{0}$, $\gamma _{j}$, the wavelength, the fractional ion to electron temperature $\sigma _{ie}$, and the atomic number of the ions $Z_{i}$. In this formalism, the far- and near-equilibrium regions are characterized by $0<\kappa _{0}<1$ ($0<\gamma _{j}<0.5$) and $\kappa _{0}>1$ ($0.5<\gamma _{j}<1$), respectively, where we analyzed the behavior of IAWs from the anti-equilibrium state towards the equilibrium state.

The summary of our results is as follows:

- The ion-sound speed varies in different thermodynamic states of the plasmas. Equivalently, the (extended/isothermal) phase speed of the IAWs relates to $\kappa _{0}$ or $\gamma _{j}$, and so it is a sensitive function of the stationary state of the plasma. Especially, the phase speed of the IAWs increases with $\kappa _{0}$ and $\gamma _{j}$ towards its maximum values in the vicinity of the isothermal states at $\gamma _{j}\rightarrow 1$ or when we have the Maxwellian distributed plasmas ($\kappa _{0}\gg 1$).
- The phase speed of the IAWs increases with the temperature of the plasma ions, as is expected from the more thermal pressure of the ions. Furthermore, it is slower for the plasmas with heavier ions.
- The (absolute value of) damping rate increases for the lower spectral indices $\kappa _{0}$, i.e. for the plasmas with more suprathermal particles. The minimum damping of IAWs occurs for a Maxwellian-distributed plasma.
- The damping rate of IAWs decreases for the longer wavelength modes. Furthermore, it tends to the minimum damping at the limit $\kappa _{0}\rightarrow \infty$ (the Maxwellian distributed plasma).
- The damping rate increases with the temperature of the plasma ions, and so the plasmas with warmer ions exhibit more prominent Landau damping.
- It was confirmed that a critical spectral index exists in the vicinity $\kappa _{0}\sim 1$ ($\gamma _{j}\sim 0.5$), in which the behavior of the IAWs diagrams is distinguished for the lower/higher values of this spectral index. The corresponding stationary state is the escape state of the plasma, where the system can escape from the far-equilibrium regions ($0<\kappa _{0}<1$ or $0<\gamma _{j}<0.5$) towards the near-equilibrium regions ($\kappa _{0}>1$ or $0.5<\gamma _{j}<1$).
- The damping time of IAWs decreases for the plasmas with more suprathermal particles (for the lower values of $\kappa _{0}$). Moreover, the damping time is considerable for the long wavelength modes.
- In the generalized KdV equation for propagation of the invariant solitary IAWs, the dispersion/nonlinear coefficient increases/decreases with $\gamma _{e}$ (towards the equilibrium states), and also they decrease with the temperature of the ions.
- On the propagation of the solitary waves, we found that: (a) Toward the equilibrium states (when $\gamma _{e}\rightarrow 1$ corresponding to the isothermal electron distribution) the maximum amplitude and the pulse width of the solitary wave increase; (b) The amplitude and the width of the solitary wave pulse decrease with the temperature of the ions; (c) The soliton pulses with more speeds have higher amplitudes and narrower width, in accordance with the classical nonlinear wave theory.

## Supplementary Information

Supplementary Information.

## Data Availability

The methods and the mathematical tools used during this study are included in the “Supplementary Material” file.
