# Supplementary material for: The invariant ion-acoustic waves in the plasma
Source: Sci Rep. 2022 Dec 16;12:21766. doi: 10.1038/s41598-022-25233-y (PMC9758181; doi:10.1038/s41598-022-25233-y)
Supplement: Supplementary file 1 — Supplementary Information. [file 41598_2022_25233_MOESM1_ESM.pdf]

Supplementary Material for “The invariant ion-acoustic waves in the plasma”

E. Saberian[1]

[1] *Department of Physics, Faculty of Basic Sciences,*
University of Neyshabur, 9319774446, Neyshabur, Iran
e.saberian@neyshabur.ac.ir

THE INVARIANT ESCORT CANONICAL DISTRIBUTION

The plasma environments in the astrophysical systems are subject to spatial and temporal long-range interactions and correlations evolving in a non-Euclidean space-time that make their behavior in non-equilibrium states. Generally, in such systems, the standard extensive Boltzmann-Gibbs-Shannon (BGS) statistics does not describe the behavior of the system exactly. There are several models for describing the phase space plasma distributions, like the kappa (κ) distribution which was introduced initially by Vasyliunas in 1968 [1] for describing plasmas out of the thermal equilibrium such as the Magnetosphere environment and the Solar winds [2], or the non-thermal model (characterized by the non-thermal parameter α) advanced by Cairns et al. in 1995 [3], which was introduced at first for explanation of the solitary electrostatic structures involving density depletions that have been observed in the upper ionosphere in the auroral zone by the Freja satellite [4], and also the celebrated q-nonextensive Tsallis model, where we review some historical developments as follows.

A suitable generalization of the BGS entropy for statistical mechanics was first proposed by Réyni [5] and subsequently by Tsallis [6]. Both Réyni and Tsallis entropies are suitable generalizations of the BGS classical entropy, however, it has been shown that the Réyni's entropic formula is not stable under several circumstances [7], but, the Tsallis entropy is stable and can appropriately provide a basis for the q-exponential distributions. The Tsallis entropy preserves the usual properties of positivity, equiprobability and irreversibility, and suitably extends the standard extensivity or additivity of the entropy to the non-extensivity. It is worth noted that the non-extensive statistical mechanics uses the concept of the q-entropy and escort probabilities. The cornerstone is the generalized entropy which was developed in decades of 70's and 80's from mathematicians. Tsallis revolutionary idea was to use this entropy in statistical mechanics. For more details about the historical background and the mathematical motivations and formulations of Tsallisnon-extensive statistics, see the chap.1 in Ref.[8].

We have to mention that the κ model in space plasmas is closely equivalent to the q-nonextensive statistics developed by Tsallis, where they may be simply connected to each other via a transformation. In fact, both the κ and q-nonextensive distributions show the deviations from the thermal equilibrium states. Especially, Livadiotis and McComas [9] examined how kappa distributions arise naturally from the Tsallis statistical mechanics.

The starting point in the non-extensive statistical mechanics is the definitions of deformed entropies, i.e., $S(q) = (q-1)^{-1}[1-\sum_k p_k^q]$, which is under the name q-Tsallis entropy [6], or equivalently $S(\kappa) = \kappa[1-\sum_k p_k^{1+\frac{1}{\kappa}}]$, where it has been formulated in terms of the κ index related to the kappa distribution [8]. Here, k numerates the spectrum of energies with (discrete) distribution p_k. These deformed entropies may reduce the celebrated Boltzmann-Gibbs-Shannon (BGS) entropy, $S = -\sum_k p_k \ln p_k$, in the asymptotic limits of $q \to 1$ or $\kappa \to \infty$.

By maximizing Tsallis q-entropy (or κ-entropy) under the constraints imposed by normalization and the energy mean value [10], we may find a primary canonical probability distribution as $p(\vec{r}, \vec{u}; T; q) \propto \left[1-(q-1)\cdot\frac{\vec{H}(\vec{r},\vec{u})}{k_B T}\right]^{\frac{1}{q-1}}$, where $\vec{H}(\vec{r},\vec{u})$ is the Hamiltonian of system. Such a canonical probability distribution, here we name it the *ordinary* distribution, was suffering from several physical problems that were solved later on by providing the *escort* probability distribution and some other constraints. The escort probability distribution P_k is constructed from the ordinary version, say p_k, via the relation $P_k = p_k^q/\Sigma_k p_k^q$ [9]. The escort probability distribution has some advantages, as opposed to the ordinary version, such as: it is independent to an energy level; it provides correct and consistent partition of the system's internal energy to the subsystem's partial internal energies; and it is consistent with a meaningful temperature (See more details in [11]).

One of the features of the spectral indices q and κ is that they are not the invariant quantities, but they depend on the number of degrees of freedom or the dimensionality of system [12, 13]. Generally, it has been proven that the difference $\kappa_d - \frac{d}{2}$ is constant and independent of d, where d is the number of degrees of freedom and κ_d is d-dimensional spectral index [12]. This shows that the indices q and κ are not invariant parameters and they depend on the dimensionality.

By considering these two fundamental constraints, i.e., the escort probability notation and the dimensional dependency of the spectral indices, the modern version of the canonical probability distribution can be written in terms of the Hamiltonian difference and d-dimensional spectral index q_d as follows

$$P(\vec{r}, \vec{u}; T; q_d) \propto \left[1+(q_d-1)\cdot\frac{\vec{H}(\vec{r},\vec{u}) - <\vec{H}(\vec{r},\vec{u})>}{k_B T}\right]^{-\frac{q_d}{q_d-1}}, \quad (1)$$

where <> denotes the ensemble average. In the absence of potential energy and by neglecting the bulk flow velocity,

we may find the canonical probability distribution as follows

$$P(\vec{u};T;q_d;d) \propto \left[1+\frac{1}{(q_d-1)^{-1}-\frac{1}{2}d}\cdot\frac{\frac{1}{2}m\vec{u}^2}{k_BT}\right]^{-\frac{q_d}{q_d-1}}. \tag{2}$$

The connection between κ and q-exponential formalism of the modern version of canonical probability distribution holds under the transformation $\kappa \equiv \frac{1}{q-1}$ or $q \equiv 1+\frac{1}{\kappa}$ [12]. So, the modern version of the canonical probability distribution can be written in terms of d-dimensional spectral index κ_d as follows:

$$P(\vec{u};T;\kappa_d;d) \propto \left[1+\frac{1}{\kappa_d-\frac{d}{2}}\cdot\frac{\frac{1}{2}m\vec{u}^2}{k_BT}\right]^{-\kappa_d-1}. \tag{3}$$

Generally, the dependent indices κ_d and q_d vary between two asymptotic limits of equilibrium and anti-equilibrium states as $\frac{d}{2}<\kappa_d<\infty$ and $1<q_d<1+\frac{2}{d}$, respectively, where the equilibrium state corresponds to $\kappa_d\to\infty$ or $q_d\to 1$, and the anti-equilibrium state corresponds to $\kappa_d\to\frac{d}{2}$ or $q_d\to 1+\frac{2}{d}$ [12]. For example, the allowed values of the spectral indices κ and q in three dimensions are given by $\frac{3}{2}<\kappa_3<\infty$ and $1<q_3<\frac{5}{3}$, respectively.

We have to emphasize that when we use the modern version of the canonical distribution as given in the generalized Eqs. (2) and (3), the equipartition of degrees of freedom holds in the same exact way as in the classical case of Maxwell-Boltzmann distributions, i.e.,

$$\frac{1}{2}m\left\langle \vec{u}^2\right\rangle = \frac{d}{2}k_BT. \tag{4}$$

It is to be noted that the presented kappa distributions are consistent with the concept of thermal equilibrium and the zeroth law of thermodynamics, so they are allowed to parameterized by temperature as the thermal distributions with a meaningful temperature [14].

By defining an invariant kappa index as of the zero dimensionality spectral index, κ_0, which is independent of the dimensionality, the degrees of freedom, or the numbers of particles, one may consider separately the physical and thermodynamic feature of the kappa index by utilizing κ_0 [12]. By noting that the d-dimensional index κ_d depends on the invariant index κ_0 by the relation $\kappa_d=\kappa_0+\frac{d}{2}$ [12], one may write the canonical probability distribution in terms of κ_0 as follows

$$P(\vec{u};\theta;\kappa_0;d) \propto \left[1+\frac{1}{\kappa_0}\cdot\frac{\vec{u}^2}{\theta^2}\right]^{-\kappa_0-1-\frac{d}{2}} \tag{5}$$

where $\theta=(\frac{2k_BT}{m})^{\frac{d}{2}}$ is the d-dimensional thermal speed of particles with mass m and temperature T. This is a multi-dimensional distribution for handling the κ or q-exponential distribution function. For example, the 1-dimensional distribution function ($d=1$) may be written in terms of κ_0 as follows

$$P(u_x;\theta;\kappa_0) \propto \left[1+\frac{1}{\kappa_0}\cdot\frac{u_x^2}{\theta^2}\right]^{-\kappa_0-\frac{3}{2}}, \tag{6}$$

where the equipartition gives $\frac{1}{2}m\left\langle u_x^2\right\rangle=\frac{1}{2}k_BT$. As we noted, the related q-exponential distributions may be derived from the simple transformation of the indices as $\kappa=\frac{1}{q-1}$, i.e., in terms of the zero dimensional index, $q_0=1+\frac{1}{\kappa_0}$, the Eq. (6) becomes

$$P(u_x;\theta;q_0) \propto \left[1+(q_0-1)\cdot\frac{u_x^2}{\theta^2}\right]^{-\frac{3q_0-1}{2(q_0-1)}}, \tag{7}$$

We may also write the other versions of the 1-dimensional distribution function in terms of κ_1, κ_2 and κ_3 or equivalently in terms of q_1, q_2 and q_3, as they have been given in Ref. [15]. In these formalisms, the formal distinction is because of the different number of degrees of freedom involved in the spectral indices.

SOME MOMENTS OF THE INVARIANT KAPPA DISTRIBUTION

After Taylor expanding of the integrands in the dielectric function of the IAWs, we have some moments in terms of the velocity derivative of the invariant kappa distribution. We have calculated these moments for deriving the Cauchy principal values in the dielectric function as follows

$$\mathcal{P}\int u_x^j \frac{\partial P_{i(e)0}}{\partial u_x}\mathrm{d}u_x = 0, \quad j = \text{even}, \tag{8a}$$

$$\mathcal{P}\int u_x \frac{\partial P_{i(e)0}}{\partial u_x}\mathrm{d}u_x = -1, \tag{8b}$$

$$\mathcal{P}\int \frac{1}{u_x}\frac{\partial P_{i(e)0}}{\partial u_x}\mathrm{d}u_x = -\frac{2}{\theta_{i(e)}^2}\cdot\left(\frac{\kappa_0+1}{\kappa_0}\right), \tag{8c}$$

$$\mathcal{P}\int u_x^3 \frac{\partial P_{i(e)0}}{\partial u_x}\mathrm{d}u_x = -\frac{3}{2}\theta_{i(e)}^2, \tag{8d}$$

where we have used the following formula given in the tables of integrals [16]

$$\int_0^\infty t^{\mu-1}(1+t^2)^{\nu-1}dt = \frac{1}{2}B(\frac{\mu}{2}, 1-\nu-\frac{\mu}{2}), \tag{9}$$

in which $B(a,b) = \frac{\Gamma(a)\cdot\Gamma(b)}{\Gamma(a)+\Gamma(b)}$ is the beta function, and $\Gamma(z)$ is the gamma function.

---

[1] V. M. Vasyliunas, J. Geophys. Res. 73, 2839 (1968).
[2] M. Maksimovic, V. Pierrard, and J. F. Lemaire, Astronomy and Astrophysics 324, 725 (1997).
[3] R. A. Cairns, A. A. Mamum, R. Bingham, R. Bostrom, R. O.Dendy, C. M. C.Nairn, and P. K. Shukla, Geophys. Res. lett. 22, 2709 (1995).
[4] P. O. Dovner, A. I. Eriksson, R. Bostrom, and B. Holback, Geophys. Res. lett. 21, 1827 (1994).
[5] A. Réyni, Acta Math. Hungarica 6, 285 (1955).
[6] C. Tsallis, J. Stat. Phys. 52, 479 (1988).
[7] S. Abe, Phys. Rev. E 66, 046134 (2002).
[8] G. Livadiotis, Kappa Distribution: Theory Applications in Plasmas (Elsevier, Netherlands, 2017).
[9] G. Livadiotis and D. J. McComes, J. Geophys. Res. 114, A11105 (2009).
[10] S. Abe, Physica A 269, 403 (1999).
[11] G. Livadiotis, J. Geophys. Res 120, 1607 (2015).
[12] G. Livadiotis and D. J. McComes, The Astrophysical Journal 88, 741 (2011).
[13] G. Livadiotis, entropy 17, 2062 (2015).
[14] G. Livadiotis, Europhys. Lett. 122, 50001 (2018).
[15] E. Saberian and G. Livadiotis, Physica A 593, 126909 (2022).
[16] Edited by M. Abramowitz and I. A. Stegun, *Handbook of Mathematical Functions* (Dover, New York, 1972).
